# Effects of Chang-Kang-Fang Formula on the Microbiota-Gut-Brain Axis in Rats With Irritable Bowel Syndrome

**DOI:** 10.3389/fphar.2022.778032

**Published:** 2022-05-09

**Authors:** Xiwen Ling, Siyuan Peng, Jingbin Zhong, Lirong Guo, Yaqin Xu, Xiaobao Jin, Fujiang Chu

**Affiliations:** ^1^ Guangdong Provincial Key Laboratory of Pharmaceutical Bioactive Substances, Guangzhou Higher Education Mega Center, Guangdong Pharmaceutical University, Guangzhou, China; ^2^ School of Traditional Chinese Medicine, Guangdong Pharmaceutical University, Guangzhou, China

**Keywords:** Chang-Kang-Fang formula, MGBA, IBS, gut microbiota, metabonomics, network pharmacology

## Abstract

Chang-Kang-Fang formula (CKF), a multi-herb traditional Chinese medicine, has been used in clinical settings to treat irritable bowel syndrome (IBS). Recent studies show that 5.0 g/kg/d CKF can alleviate the symptoms of IBS rats by modulating the brain-gut axis through the production of brain-gut peptides (BGPs), thus relieving pain, and reversing the effects of intestinal propulsion disorders. However, the exact mechanisms underlying the therapeutic effects of CKF in IBS remain unclear. The microbiota-gut-brain axis (MGBA) is central to the pathogenesis of IBS, regulating BGPs, depression-like behaviors, and gut microbiota. Given that CKF ameliorates IBS *via* the MGBA, we performed metabolomic analyses, evaluated the gut microbiota, and system pharmacology to elucidate the mechanisms of action of CKF. The results of intestinal tract motility, abdominal withdrawal reflex (AWR), sucrose preference test (SPT), and the forced swimming test (FST) showed that the male Sprague–Dawley rats subjected to chronic acute combining stress (CACS) for 22 days exhibited altered intestinal motility, visceral hypersensitivity, and depression-like behaviors. Treatment of IBS rats with CKF normalized dysfunctions of CACS-induced central and peripheral nervous system. CKF regulated BDNF and 5-HT levels in the colon and hippocampus as well as the expressions of the related BGP pathway genes. Moreover, the system pharmacology assays were used to assess the physiological targets involved in the action of CKF, with results suggesting that CKF putatively functioned through the 5-HT-PKA-CREB-BDNF pathway. LC-MS-based metabolomics identified the significantly altered 5-HT pathway-related metabolites in the CKF treatment group, and thus, the CKF-related signaling pathways were further examined. After pyrosequencing-based analysis of bacterial 16S rRNA (V3 + V4 region) using rat feces, the Lefse analysis of gut microbiota suggested that CKF treatment could induce structural changes in the gut microbiota, thereby regulating it by decreasing *Clostridiales*, and the F-B ratio while increasing the levels of *Lactobacillus*. Furthermore, the integrated analysis showed a correlation of CKF-associated microbes with metabolites. These findings showed that CKF effectively alleviated IBS, which was associated with the altered features of the metabolite profiles and the gut microbiota through a bidirectional communication along the microbiota-gut-brain axis.

## Introduction

Irritable bowel syndrome (IBS) is characterized by abdominal pain or abdominal discomfort, changes in stool shape or bowel habits, and mental symptoms of depression and anxiety (Chen et al., 2014). However, it lacks a form to explain the symptoms and the chronic functionality of biochemical indicators of intestinal diseases (Yu et al., 2015; Chen et al., 2017). The global overall prevalence rate of IBS is 5%–20%; it is 9%–22% in the west and 4%–10% in the Asian countries (Vaiopoulou et al., 2014; Moraru et al., 2015). To date, there is no elaborate diagnostic strategy for IBS due to the lack of symptoms of an organic disease. Notably, the diagnosis can only be confirmed through a series of examinations to rule out inflammation, gastrointestinal infection, and organic malignancies. The pathogenesis of IBS is complex and is associated with visceral sensory allergies, abnormalities in gastrointestinal motility, intestinal microflora, overuse of small intestinal bacteria, immune abnormalities, psychosocial factors, and abnormalities in the brain-enteral axis. Owing to the rapid development in detection technology in recent years, intestinal microflora in various gastrointestinal diseases has emerged as a research hotspot. Thus, intestinal flora has gradually gained recognition as a vital factor underlying the pathogenesis of IBS. Notably, disorders of the intestinal flora are common when the body is influenced by food, climate, environment, and other factors, thereby leading to the emergence of gastrointestinal diseases including IBS.

Metabolomics uses modern high-throughput analysis techniques for a holistic qualitative and quantitative assessment of metabolites produced within a cell or by an organism at a given period, such as upon drug stimulation or in different pathological states. Metabolomics requires multi-variable dimensional reduction statistics to construct and systematically analyze the physiological and pathological states, and their relationship with environmental factors and gene composition. The research does not consider people as an isolated system, but as belonging to a global network involving the environment, flora, and intestinal individual differences. Previous studies on serum metabolomics of patients from IBS show changes in multiple metabolite contents (Elia et al., 2008; Zeber-Lubecka et al., 2016).

According to “Jingyue Quanshu” from the Ming Dynasty, Chang-Kang-Fang, a multi-herb Chinese medicinal formula, has been used in China for the clinical treatment of IBS (Mao et al., 2017; Fan et al., 2021). This formula is composed of *Salvia miltiorrhiza Bunge* (Lamiaceae; *Salviae miltiorrhizae* radix et rhizoma); *Paeoniae Alba Radix* (Paeoniaceae; the root of *Paeonia lactiflflora* Pall.) (15 g), *Fagopyri Dibotryis Rhizoma* [Polygonaceae; the rhizome of Fagopyrum cymosum (Trevir.) Meisn.] (8 g), *Saposhnikoviae Radix* [Apiaceae; the root of *Saposhnikovia divaricata* (Turcz.) Schischk.] (6 g), *Cuscutae Semen* (Convolvulaceae, the seed of *Cuscuta chinensis* Lam.) (5 g), *Rehmanniae Radix* [Orobanchaceae, the root of *Rehmannia glutinosa* (Gaertn.) DC.] (5 g), *Coptidis Rhizoma* (Ranunculaceae, the rhizome of *Coptis chinensis* Franch.) (3 g), and *Periostracum cicadae* (Cicadidae, the exuviae of *Cryptotympana pustulata* Fabricius.) (3 g) (Mao et al., 2017). The equivalent dose was calculated based on the recommended dosage for humans with the conversion coefficient of 7.4 and the daily dose was approximately 5.0 g/kg (Nair and Jacob, 2016). In a clinical trial, patients with IBS were divided into groups in a randomized manner and administered either CKF or trimebutine maleate (TM) for 12 weeks. Therapeutic responses were observed in 85.2% of the CKF-treated patients and 64.7% in TM-treated patients (Cao and Lu, 2014). A recent study shows that 5.0 g/kg/d CKF can alleviate intestinal motility disorders by regulating the production of brain-gut peptides (BGPs), which in turn regulate the brain-gut axis and reduce symptoms of IBS in rats (Mao et al., 2017). Having an efficacy likewise, natural plant drugs have safer and less adverse effects as compared to synthetic chemical drugs. 5.0 g/kg/d CKF yields a significant improvement to the degree similar to that of the western medicine, trimebutine maleate (TM) control group in a rat model of IBS (Mao et al., 2017). However, the authors of this study did not elucidate the mechanisms underlying the therapeutic effects of CKF in IBS encompassing the modulation of the brain-gut axis.

Network pharmaceutical research is based on the principles of network theory and systems biology (Hopkins, 2008; Zhang et al., 2019). The concept of network pharmacology is based on multiple nodes in a targeted interconnected molecular system rather than a single molecule, thus, it shows better curative effects and fewer side effects (Ashburner et al., 2000). The signaling protein exerts a biological function and interacts with its partners, which can be analyzed by the Genome Project gene ontology (GO) enrichment analysis biological functions (Janga and Tzakos, 2009; Zhang et al., 2012; Gao et al., 2018). This is in line with the multi-component and multi-targeted theory of Chinese medicine. Researchers have now increasingly been using network pharmacology to predict the main active ingredients and potential target groups of traditional Chinese medicine and determine their mechanisms of action (Ren et al., 2014).

The purpose of this study was to investigate the *in vivo* effects of CKF in alleviating IBS and depression-like behavior caused due to chronic acute combining stress (CACS) (Yu et al., 2019). The findings verified the strong efficacy of the model, which also provided insights into the pathological process as well as the vulnerability and trigger factors of IBS. Treatment with CKF could significantly improve the symptoms of the central and peripheral nervous system and was related to the differential levels of 5-HT and BDNF in the hippocampus and colon. We examined the relationship between the microbiota-gut-brain axis (MGBA) and CKF treatment. Herein, we studied the effects of CKF on MGBA in IBS and discussed the protective roles of CKF on IBS through the 5-HT-PKA-CREB-BDNF signaling pathway. We also evaluated the effects of CKF on the characteristics of the gut microbiota during IBS remission. In addition, network pharmacology and metabolomics were performed to understand the multi-target regulation of the 5-HT level and the potential mechanism and signal pathway underlying the effects of CKF against IBS.

## Materials and Methods

### Preparation of Chang-Kang-Fang Formula

The herbal constituents of CKF were purchased from the Guangdong Province Traditional Chinese Medical Hospital (Guangzhou, China), were morphologically authenticated by Prof. H. Y. Ma following the standards of China Pharmacopoeia (Part I, 2020 Version). A voucher number was assigned to each specimen and preserved in the School of Traditional Chinese Medicine, Guangdong Pharmaceutical University. The procedure for synthesizing the CKF extract has been described previously (Mao et al., 2017). Briefly, the seven herbal components of CKF (150 g *Paeoniae Alba Radix*, 60 g *Saposhnikoviae Radix*, 50 g *Rehmanniae Radix*, 50 g *Cuscutae Semen*, 30 g *Coptidis Rhizoma*, 80 g *Fagopyri Dibotryis Rhizoma*, and 30 g *Periostracum cicadae*) were blended for 2 h in 3-volumes of water (1,350 ml). The mixture was filtered and the filtrate was collected (Mao et al., 2017). Finally, the liquid was condensed and concentrated to 450 ml (1 g/ml) and stored at 4°C until subsequent use. For animal treatment, the solution was filtered and concentrated in a vacuum at 50°C. The final concentration was 5 g crude drug per ml. The resulting extract was stored at −20°C. The CKF was performed in previous study to simultaneously identify the major chemical components and the UPLC-Q-TOF-MS chromatogram of CKF obtained from its water extract is shown in Supplementary Figure S1 (Supplementary Table S1) (Mao et al., 2017).

### Animals and Experimental Design

A total of 32 (6-weeks old, 180–200 g body weight) male Sprague-Dawley (SD) rats were obtained from the Centre for Experimental Animals, Guangdong Province [Guangzhou, China approval number SCXK (Yue) 2017-0002]. Rats were housed in controlled conditions (room temperature: 23 ± 2°C; relative humidity: 55 ± 5%, and 12-h dark/12-h light cycle) for 1 week with ad libitum access to food and water. The animal studies were conducted following the guidelines for the Care and Use of Experimental Animals. The designs of all animal experiments were approved by the Guangdong Pharmaceutical University Animal Care and Use Committee, China [SCXK (Yue) 2017-0125].

### Establishing the Rat Model of Irritable Bowel Syndrome

**FIGURE 1:**
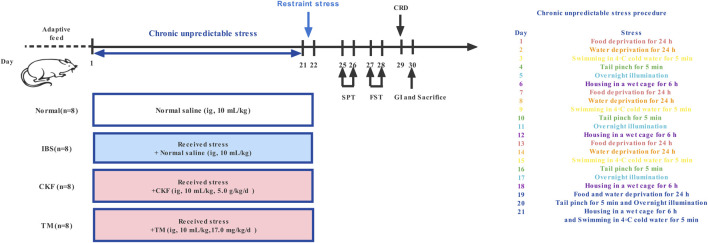
Timeline of experimental procedures.

CACS-treated rats were subjected to chronic unpredictable stress for 21 days, as illustrated in Figure 1 (Zou et al., 2008; Yu et al., 2015). On day 22, these rats were subjected to restraint stress for 3 h. The stool samples of the rats were collected within a 4 h period and the fecal water content and total fecal output were calculated. After weighing (wet weight), the stool samples were dried under vacuum overnight and the dry weight was recorded (Nelson et al., 2011). To verify the IBS rat model, visceral hypersensitivity was measured using the abdominal withdrawal reflex (AWR) scoring system; depression-like behaviors were measured using the sucrose preference and forced swimming tests; upper GI transit was used to evaluate gastrointestinal motility, and the number of fecal pellets and fecal water content was used to evaluate the severity of diarrhea in each group as described previously (Li et al., 2018; Xu et al., 2018).

### Grouping and Treatment

After acclimatization, the rats were randomly divided into the following four groups (*n* = 8): Normal, IBS, CKF, and trimebutine maleate (TM) (Figure 1). TM was purchased from Shanghai yuanye Bio-Technology Co., Ltd. (Shanghai, China). The rats in the normal and IBS groups were intragastrically administered 10 ml/kg normal saline, while those in the CKF group rats were intragastrically administered 5.0 g/kg CKF, while in the TM treated group (10 ml/kg body weight), 17.0 mg/kg TM was administered. These treated rats were regularly examined and changes in the characteristics of food intake and body weights were recorded.

### Assessment of Gastrointestinal Motility

Rats in each group were subjected to fasting for 24 h, followed by an oral administration of 2 ml suspension of the charcoal meal (10% charcoal in 5% gum arabic) (Lv et al., 2011). These rats were sacrificed after 20 min. Next, the abdomen was opened up and the intestines from the pyloric junction to the caecal end were quickly excised (Dong et al., 2014). After dissection, the entire length of the intestines from the pyloric junction to the caecal end and the distance traveled by the charcoal meal were measured. The percentage of the distance traveled by the charcoal meal to the total length of the pyloric junction to the caecal end was the measure of the upper GI transit (Dong et al., 2014).

### Assessment of Abdominal Withdrawal Reflex

The visceral sensitivity to colorectal distention (CRD) was evaluated by measuring the AWR following a previously described protocol (Al-Chaer et al., 2000; Winston et al., 2007). Before the procedure, rats were allowed free access for drinking but were food-deprived for 24 h. Next, the rats were lightly anesthetized with 2% isoflurane, followed by insertion of an 8F catheter through the anus after lubrication with paraffin oil. Throughout, we ensured, by taping the associated tubing onto the rat tail, that 1 cm of a tube was left between the end of the gasbag and anus (Yu et al., 2015; Gong et al., 2015; Xu et al., 2019). Rats were subsequently placed in Lucite cubicles (20 cm × 8 cm × 8 cm) and allowed to acclimatize to the environment for 20 min (after awakening) before the test (Babygirija et al., 2017). After acclimatization, the rats were subjected to distention pressures (10, 20, 30, and 40 mmHg) for 20 s with a 5-min interval between different pressures. Finally, the AWR scores were determined according to as detailed in Table 1 (Yu et al., 2019; Li et al., 2020).

**TABLE 1:** Criteria for AWR scoring.

Score	Score behavioral Responses to CRD
0	No behavioral response
1	Occasionally twist their heads
2	Abdominal muscles contracted slightly
3	Lifting of abdomen
4	Body arching and lifting off the ground

### Sucrose Preference Test

Sucrose preference test was performed by providing the rats with both 1% sucrose solution and double distilled water for 2 days (Wang et al., 2015). Briefly, rats were individually housed and were food- and water-deprived for 24 h (Nadal et al., 1996). On the following day, rats were free to choose between water and 1% sucrose solution. The sucrose preference was determined according to the ratio of the sugar solution consumed to that of the water (water and sucrose solution).

### Forced Swimming Test

Rats were subjected to 15 min of training for forced swimming in a glass cylinder 24 h before the formal test (height = 45 cm and diameter = 25 cm) at a water depth of 30 cm and temperature of 24 ± 1°C. After administration, rats were again placed in the glass cylinder for swimming for 5 min, and the immobility time within 5 min was observed and recorded (Rao et al., 2021). Immobility time was defined as the time when the rat stopped struggling, floated in the water, and remained motionless, or only made necessary light movements to keep its head afloat in the water (Porsolt et al., 1977; Pan et al., 2021).

### Enzyme-Linked Immunosorbent Assay

The distal colon and hippocampal tissues were isolated and homogenized in cold PBS solution. Next, the frozen colonic and hippocampal tissues were homogenized and lysed in tissue lysis buffer, followed by centrifugation at 12,000 g for 10 min at 4°C. The supernatant was collected and the levels of 5-HT and BDNF in the colonic and hippocampal tissues were measured using ELISA kits following the manufacturer’s instructions (Shanghai MLBIO Biotechnology Co., Ltd.).

### Detection by Real-Time Quantitative PCR

Total RNA was extracted from the distal colon and hippocampal samples using the Trizol reagent (Accuratse Biology Co., Ltd., China), followed by cDNA synthesis using the PrimeScriptTM RT reagent Kit with a gDNA Eraser (Accuratse Biology Co., Ltd., China) according to the manufacturer’s instructions. Next, the mRNA levels of individual genes were determined through real-time PCR using the 2 × SYBR Green Pro Taq HS premix (Accuratse Biology Co., Ltd., China) on the CFX Connect fluorescence quantitative PCR detection platform (BIO-RAD, United States). The 20 µl PCR reaction mixture comprised of the following components: 10 µl 2 × SYBR Green Pro Taq HS Premix, 0.4 µl forward primer (10 µM), 0.4 µl reverse primer (10 µM), 2 µl reaction solution (cDNA), and 7.2 µl RNase free water. The standard protocol for a shuttle PCR was as follows: stage 1 included one cycle of initial denaturation at 95°C for 30 s; stage 2 included 40 cycles of the PCR at 95°C for 5 s and 60°C for 30 s, and stage 3 was the dissociation step. The data were evaluated according to the comparative threshold cycle (Cq) value. Finally, the relative levels of mRNA expression of 5-HT1A, PKA, CREB, and BDNF were normalized against that of glyceraldehyde-3-phosphate dehydrogenase (GAPDH) mRNA and calculated using the 2^−∆∆CT^ method. The primers used in this experiment as listed in Table 2.

**TABLE 2:** Primer sequences for rat target genes.

Gene name	Primer sequence (from 5′ end to 3′ end)	Product size (bp)
Gapdh_1F	ATG​GCT​ACA​GCA​ACA​GGG​T	189
Gapdh_1R	TTATGGGGTCTGGGATGG	
Bdnf_2F	CTC​TGC​TCT​TTC​TGC​TGG​A	144
Bdnf_2R	TATCTGCCGCTGTGACC	
Htr1a_4F	GGGCAACTCCAAAGAGCA	140
Htr1a_4R	TCA​CCG​TCT​TCC​TTT​CAC​G	
Creb1_4F	GCC​ACA​ACC​AGA​AAG​ACA​A	150
Creb1_4R	TGAGGGCAGAAGTGGAAG	
Prkaca_4F	AAGGAAGGAACTGGGCTT	169
Prkaca_4R	CACGGCAAGAGGTGATG	

### Immunohistochemistry

Rats were sacrificed at the end of the experiments. Brain and colon tissues were then isolated, embedded on paraffinized blocks, and cut into 7 and 4 µm thick sections, respectively, using a microtome (Leica, Germany). Next, the sections were incubated with the anti-5-HT1A rabbit polyclonal antibody (Cat. No.D260012), anti PKA rabbit polyclonal antibody (Cat. NO.D164496), anti-CREB1 (Phospho-Ser133) rabbit polyclonal antibody (Cat. No.D155349), and anti-BDNF polyclonal antibody (Cat. No.D221057) (1:50) (Sangon Biotech, China), overnight at 4°C in a dilution ratio of 1:100 using the BondTM Primary Antibody Diluent (Servicebio, China). On the next day, the sections were incubated for 1 h with horseradish peroxidase 4-layered goat anti-rabbit secondary antibodies at 37°C (Sangon Biotech, China) according to the manufacturer’s instructions. Finally, the sections were treated with diaminobenzidine (DAB) solution (Servicebio, China) and visualized under a microscope (NIKON, Eclipse, Ci). We measured the integrated optical density (IOD) from at least three fields of each slice using the Image pro-plus 6.0 software, which could accurately reflect the complete expression of the proteins in immunohistochemical staining.

### Analysis of the Gut Microbiota

Three fecal samples were randomly collected from rats in the Normal, IBS, and CKF groups at the end of the therapy cycle for further 16S rDNA gene sequencing. Rats were sacrificed and the fresh stool samples from the colon were collected and immediately stored at −80°C until further analyses. Subsequently, bacterial genomic DNA was extracted from the frozen stool samples using the Qiagen QIAamp DNA stool Mini Kit (Hilden, Germany) according to the manufacturer’s instructions. Next, conventional PCR was performed to amplify the 16S rRNA in the V3-V4 region (341F-805R, F: GAT​CCT​ACG​GGA​GGC​AGC​A; R: GCT​TAC​CGC​GGC​TGC​TGG​C). Following were the PCR conditions: initial denaturation at 98°C for 1 min, followed by 30 cycles of denaturation at 98°C for 10 s, annealing at 50°C for 30 s, and elongation at 72°C for 60 s with a final hold at 72°C for 5 min. Samples were then purified using the MinElute Gel Extraction Kit (Qiagen, China), and samples with lengths of 400–450 bps were chosen for further experiments. Next, sequencing libraries were generated using the NEB Next Ultra DNA Library Prep Kit from Illumina (NEB, United States) following the manufacturer’s protocol. After the addition of the index codes, high-throughput pyrosequencing was performed for the PCR products on the Illumina MiSeq/HiSeq2500 platform (Biomarker Technologies Co., Ltd., Beijing, China). The QIIME software package (18.0 version) was employed to identify the operational taxonomic units (OTUs) and the RDP classifier was used to annotate the taxonomic information for each representative sequence using the Greengenes database (13.5 version). The weighted UniFrac distance-based principal coordinate analysis (PCoA) and binary Jaccard pair group method with arithmetic mean (UPGMA) clustering were performed using the QIIME software package. Finally, to identify the biomarker species in different groups, the linear discriminant analysis effect size (LEfSe) was estimated using the Metastats software. PICRUSt (Phylogenetic Investigation of Communities by Reconstruction of Unobserved States, http://picrust.github.io/picrust/) is a bioinformatic software package designed to predict the functional profiling of microbial communities based on the 16S rDNA sequences (Langille et al., 2013). STAMP (http://kiwi.cs.dal.ca/Software/STAMP) was used for functional profiling (Park et al., 2013).

### Serum Metabolomics

Methanol (400 µl) was added to 100 µl of the serum sample, followed by vortexing for 60 s and centrifugation at 12,000 rpm for 10 min at 4°C. Subsequently, the supernatant was transferred into a fresh 20 µl vial for UPLC-MS analysis (Liu et al., 2018). The protocol followed is described in Supplementary Methods.

### Systematic Evaluation of the Mechanisms of Action

CKF comprises the following seven botanical drugs: *Paeoniae alba Radix., Fagopyri dibotryis Rhizoma.*, *Saposhnikoviae radix*, *Cuscutae semen*, *Rehmanniae radix*, *Coptidis rhizoma*, *and Periostracum cicadae* (Fan et al., 2021). First, we performed an extensive literature review for these botanical drugs using the Traditional Chinese Medicine Systems Pharmacology Database and Analysis Platform (TCMSP) and retrieved the information on all chemical ingredients and their protein targets (https://sea.bkslab.org/). The molecules that met the criteria of bioavailability (OB) ≥30% and drug-likeness (DL) ≥0.1 (Xu et al., 2012) were used for further analysis. Second, the targets were identified after ascertaining the molecular targets associated with IBS and depression and were utilized for further analysis. Third, an interaction network was constructed for the identified targets using the STRING database (https://string-db.org/) to systematically elucidate the mechanism underlying the effects of CKF(3). Fourth, to explain the potential role of the active components in CKF in gene function and signaling pathways in the rat model, gene ontology (GO) enrichment and Kyoto Encyclopedia of Genes and Genomes (KEGG) pathway analyses were performed for the hub genes using the Database for Annotation, Visualization and Integrated Discovery (DAVID, https://david.ncifcrf.gov/summary.jsp) tools for functional enrichment analysis (Chen et al., 2021). Species and background were limited to *Rattus norvegicus* (rat). The results were then plotted using the tools in Bioinformatics Web Server (http://www.bioinformatics.com.cn), an online platform for data analysis and visualization. Moreover, the gene-pathway network was constructed to screen the key genes targeted by CKF and relevant in the treatment of IBS and depression. To show the relationship among CKF active components, the hub targets, and KEGG pathways, a Sankey diagram was constructed using the tools of Bioinformatics Web Server (http://www.bioinformatics.com.cn).

### Statistical Analysis

All statistical analyses were performed using the SPSS Statistics 17.0 software, and all data were presented as mean ± SD. One-way analysis of variance (ANOVA) was used for multiple comparisons. *p* < 0.05 was considered statistically significant. To demonstrate the relationships between parameters, Spearman’s correlation was measured. The correlation coefficient was always in the range of +1 to −1. The closer was the absolute value of the correlation coefficient to 1, the closer was the relationship to being perfect linear.

## Results

### Chang-Kang-Fang Formula Ameliorates Weight Loss, the Occurrence of Diarrhea, and Intestinal Dysfunction in Rats With Irritable Bowel Syndrome

**FIGURE 2:**
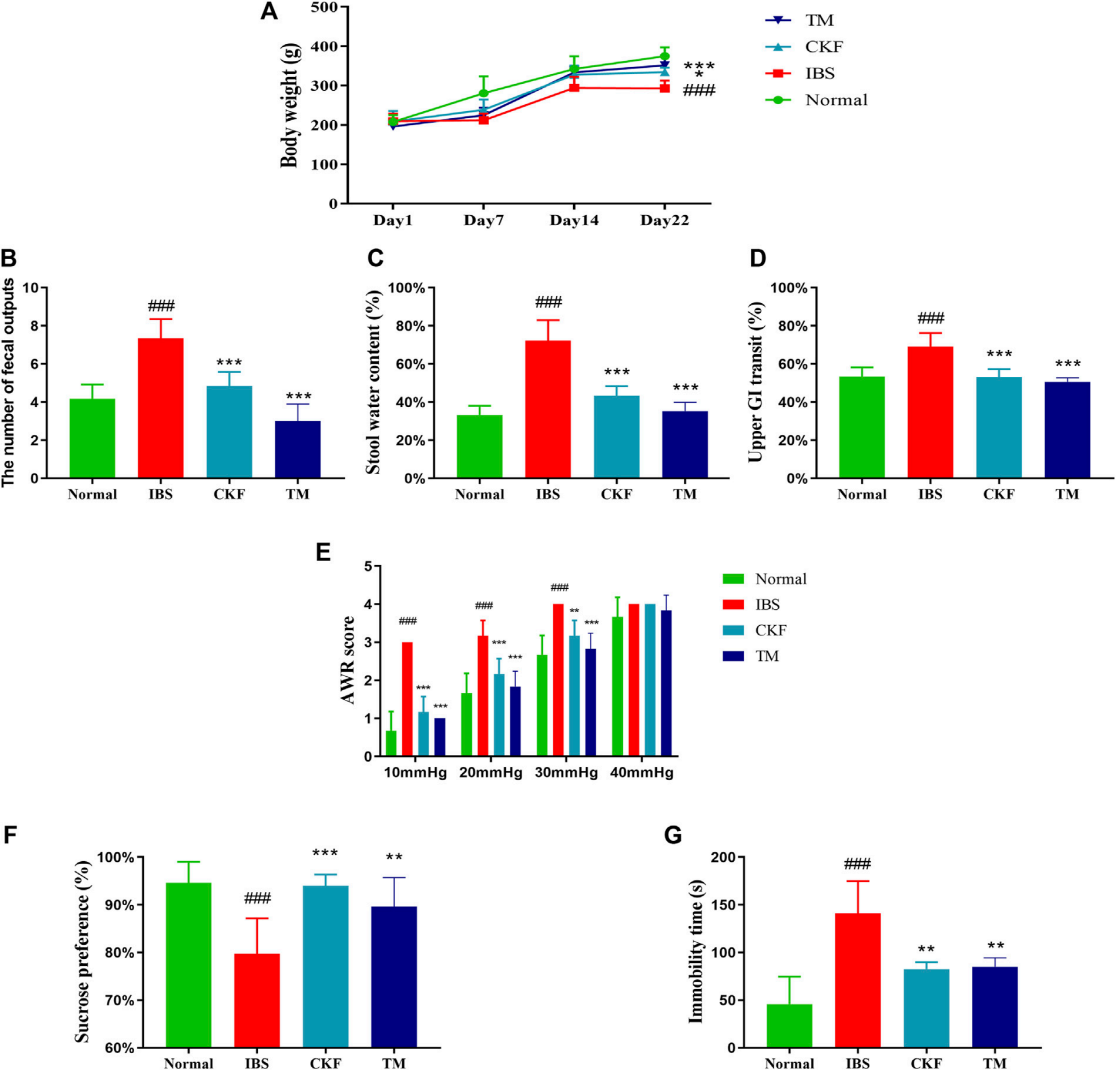
Chang-Kang-Fang formula affects the body weight, the occurrence of diarrhea, intestinal tract motility, abdominal withdrawal reflex (AWR), the sucrose preference test (SPT), and forced swimming test (FST) in IBS rats **(A)** The changes in the body weights of rats. **(B)** Fecal output **(C)** stool water content **(D)** Intestinal tract motility. **(E)** AWR scores. **(F)** Percentage of sucrose water consumed in the SPT. **(G)** The immobility time of rats in FST. Values are presented as the means ± SD (*n* = 8). ^###^ *p* < 0.001 compared to Normal, *p < 0.05, **p < 0.01 and ***p < 0.001 compared to IBS.

After modeling IBS in rats, the body weights in the IBS group were found to be substantially lower than in those in the normal group (*p* < 0.001). The initial body weight and food intake were not statistically different between the IBS, CKF, and TM groups on day 1. After administration of CKF and TM for 22 days, the body weights in the CKF and TM groups increased significantly relative to the IBS group (*p* < 0.05, *p* < 0.001, Figure 2A).

**FIGURE 3:**
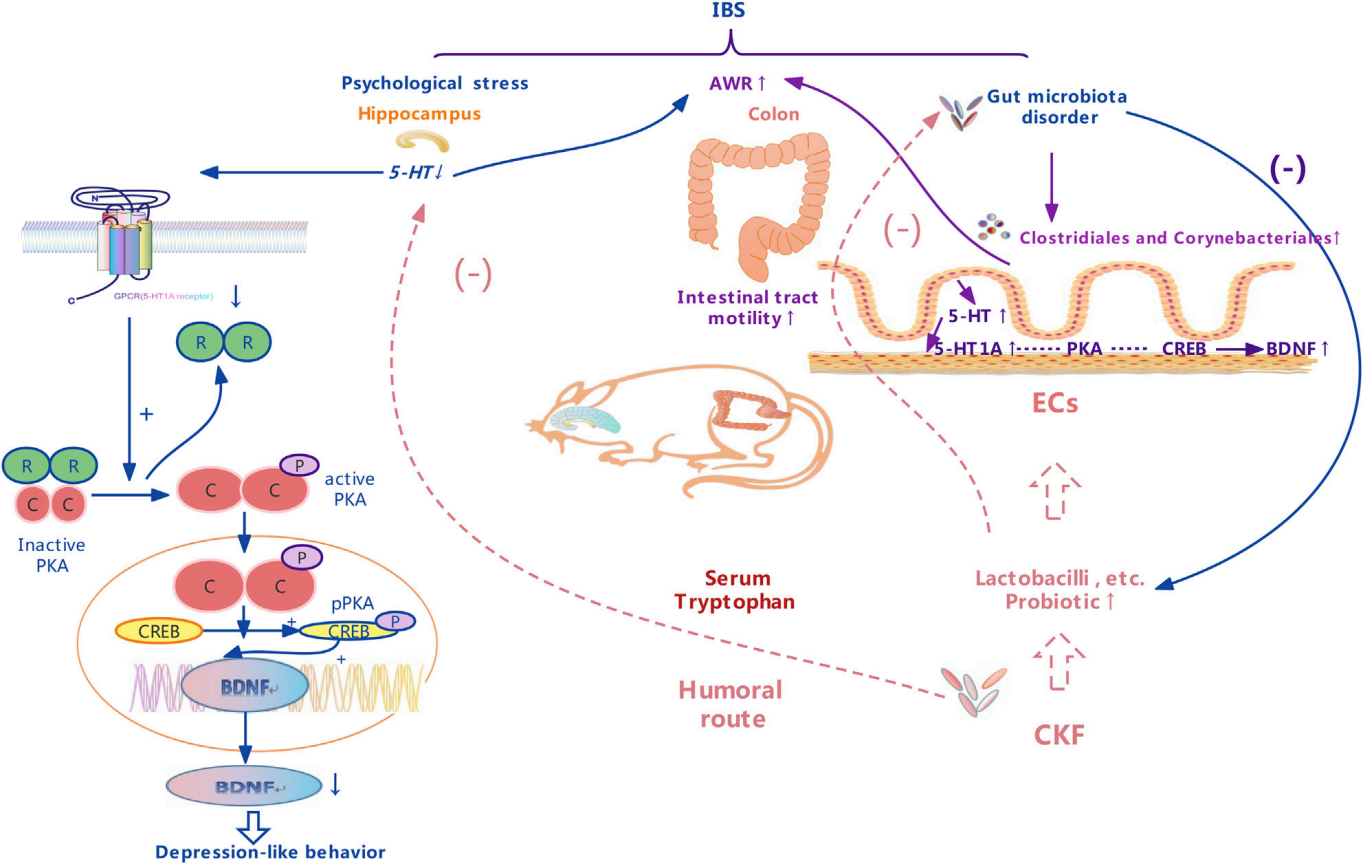
A schematic representation of therapeutic effects of the Chang-Kang-Fang formula on the microbiota-gut-brain axis.

We also measured the total fecal output and the water content in the stool for 4 h (Figure 3). The results showed that there was an increase in total fecal output and the stool water content in the IBS as compared to the normal rats (*p* < 0.001, Figures 2B,C). Moreover, the total fecal output and the water content in stool samples of rats in the CKF and TM groups decreased markedly as compared to the rats in the IBS group (*p* < 0.001, *p* < 0.001, Figures 2B,C).

The time for GI transit was used to evaluate intestinal mobility in rats. The results suggested that there was an increase in the GI transit rate in rats in the IBS group as compared to those in the normal group (*p* < 0.001, Figure 2D). This indicated that the frequency of GI transit increased significantly after the modeling of IBS. Moreover, rats in the CKF and TM groups exhibited decreased upper GI transit as compared to those in the IBS group (*p* < 0.001, *p* < 0.001).

### Chang-Kang-Fang Formula Alleviates Stress-Induced Depression-Like Behavior and Visceral Hypersensitivity in Rats With Irritable Bowel Syndrome

There was a significant increase in the AWR scores in the IBS group as compared to the normal group, which indicated increased visceral sensitivity after the modeling of IBS. However, the AWR scores decreased significantly after CKF and TM treatments as compared to that in the IBS rats (Figure 2E), which suggested an improved visceral sensitivity after treatment with CKF and TM.

The antidepressant-like effects of CKF were assessed by sugar preference and forced swimming tests. The IBS group showed a significant decrease in the sucrose preference ratio (*p* < 0.001, Figure 2F). Treatment with CKF and TM significantly increased the sucrose preference ratio as compared to the IBS group (*p* < 0.001, *p* < 0.01, Figure 2F). Results of the forced swimming test showed that rats with IBS for 3 weeks had a longer period of immobility as compared to those in the control group (*p* < 0.001, Figure 2G). In addition, rats treated with CKF (5 mg/kg) and TM showed decreased immobility time as compared to those in the IBS group (*p* < 0.01, *p* < 0.01, Figure 2E). Collectively, these results suggested that CKF exhibited anti-depressant-like effects in the rat model of IBS.

### Gene and Protein Expressions of 5-HT and BDNF in Irritable Bowel Syndrome Rats Treated With Chang-Kang-Fang

**FIGURE 4:**
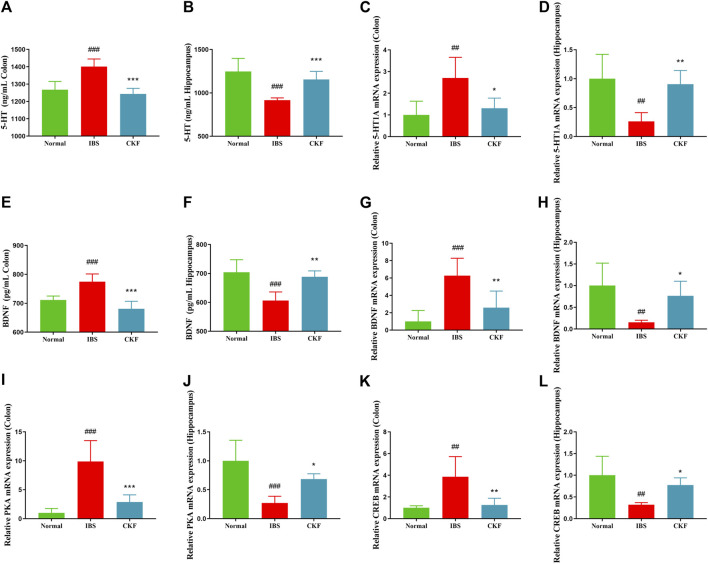
Chang-Kang-Fang formula affects the levels of 5-HT, 5-HT1AR, BDNF, PKA, and CREB in IBS rats. **(A,B)** 5-HT levels in colon and hippocampus. **(C,D)** The relative expression of 5-HT1AR in colon and hippocampus. **(E,F)** The relative expression of BDNF in colon and hippocampus. **(G,H)** The relative expression of BDNF in colon and hippocampus. **(I,J)** The relative expression of PKA in colon and hippocampus. **(K,L)** The relative expression of CREB in colon and hippocampus. Values are presented as the means ± SD (*n* = 6). ^##^ *p* < 0.01, ^###^ *p* < 0.001 compared to Normal, *p < 0.05, **p < 0.01 and ***p < 0.001 compared to IBS.

**FIGURE 5:**
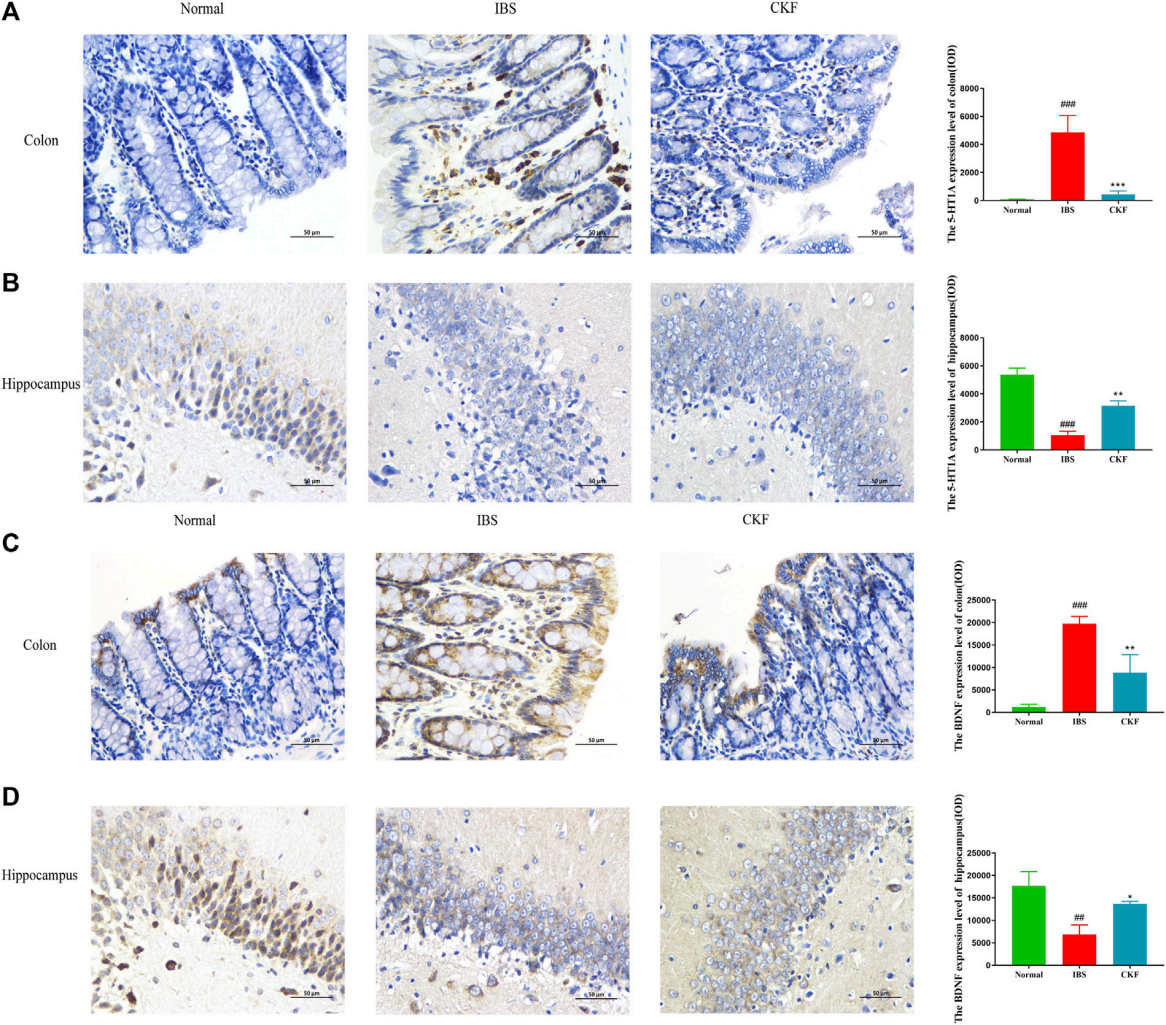
Immunohistochemical staining in the colon and hippocampal **(A,B)** 5-HT1AR and **(C,D)** BDNF. Values are presented as the means ± SD (*n* = 3). ^###^ *p* < 0.001 compared to Normal,*p < 0.05, **p < 0.01 and ***p < 0.001 compared to IBS.

To examine the efficacy of CKF in IBS rats, the expression levels of 5-HT and BDNF in the colon and hippocampal tissues were determined, and the findings are shown in Figures 4A,B,E,F. The results showed that the concentration of 5-HT and BDNF in colon tissues decreased significantly after CKF treatment as compared to the IBS group, whereas 5-HT and BDNF levels were markedly high in the hippocampal tissues after CKF treatment. To determine the levels of mRNA expression of 5-HT1A and BDNF, qPCR analysis was performed. The results showed that the levels of expression of both genes were higher in colon tissues, while lower in hippocampus tissues in the IBS group as compared to the normal group (Figures 4C,D,G,H). However, CKF treatment caused a significant decrease in the expressions of 5-HT1A and BDNF in the colon (Figures 4C,G), however, those in the hippocampus were upregulated (Figures 4D,H). Next, the immunohistological changes in the protein levels of 5-HT1A and BDNF were examined in the colon. The results suggested that stress caused an increase in the levels of 5-HT1A and BDNF on the membrane surface of the intestinal tissues, which decreased substantially after CKF treatment (Figures 5A,C). Furthermore, potential immunohistological changes in the protein expressions of 5-HT1A and BDNF in the dentate gyrus (DG) of the hippocampus were evaluated. The results showed a significant increase in 5-HT1A and BDNF-positive protein expression in the DG of the hippocampus in the CKF group relative to the IBS group (Figures 5B,D).

### Gene and Protein Expressions of PKA and CREB in Irritable Bowel Syndrome Rats Treated With Chang-Kang-Fang Formula

**FIGURE 6:**
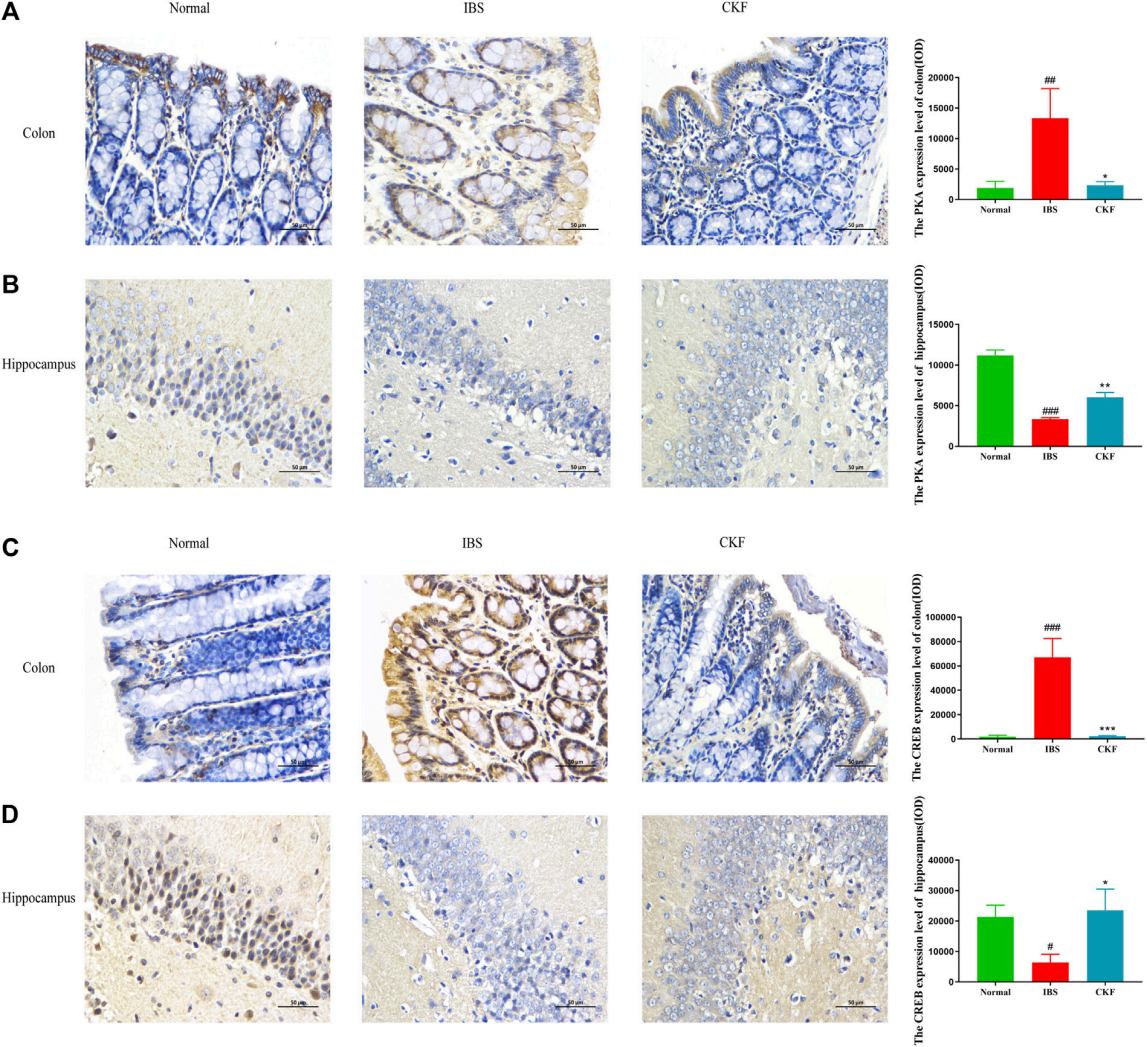
Immunohistochemical staining for the colon and hippocampal **(A,B)** PKA and **(C,D)** CREB. Values are presented as the means ± SD (*n* = 3). ^###^ *p* < 0.001 as compared to Normal,*p < 0.05, **p < 0.01 and ***p < 0.001 as compared to IBS.

Using qPCR analysis, we examined the gene expressions of PKA and CREB. The results showed higher levels of PKA and CREB expression in colon tissues; the expression of both genes in the hippocampal tissues of rats in the IBS group reduced as compared to those in the normal group (Figure 4). However, CKF treatment caused a decline in the expression of PKA and CREB in colon tissues (Figures 4I,K), and an increase in the hippocampi (Figures 4J,L). Next, we examined the immunohistological changes in protein levels of PKA and CREB in the colon. The results showed that stress caused an increase in PKA and CREB density on the membrane surface of the intestinal tissues, which decreased after CKF treatment (Figures 6A,C). Furthermore, potential immunohistological changes in the protein levels of PKA and CREB in the DG of the hippocampus were examined. Results showed a significant increase in PKA and CREB-positive protein in the DG of hippocampus tissues obtained from rats in the CKF group as compared to those in the IBS group (Figures 6B,D).

### Chang-Kang-Fang Formula Modulates Gut Microbiota in Irritable Bowel Syndrome Rats

**FIGURE 7:**
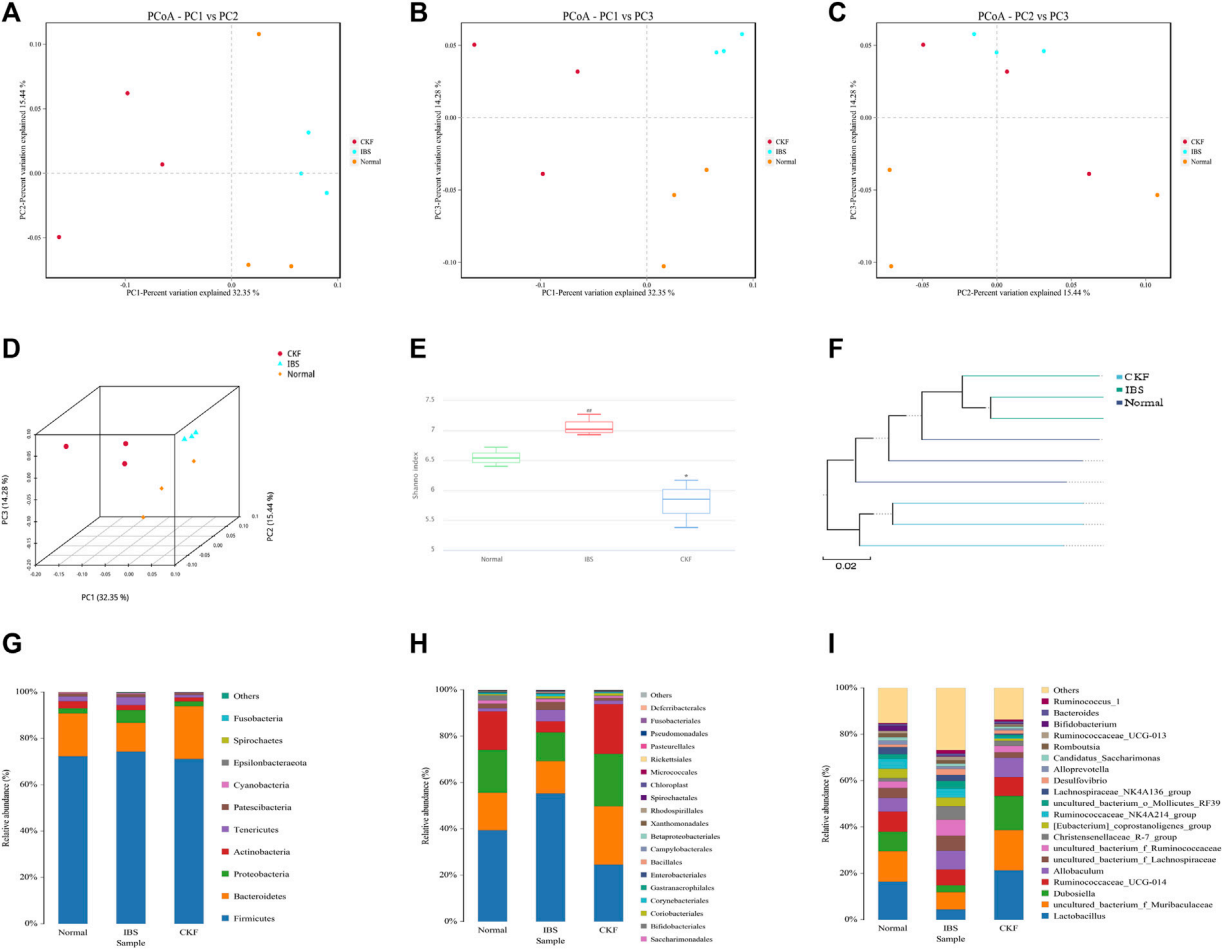
Responses of the diversity, richness, and structure of the gut microbiota to CKF in IBS rats. **(A–D)** Binary Jaccard-based PCoA. **(E)** Shannon index. **(F)** Binary Jaccard cluster tree based on UPGMA. **(G–I)** Relative abundances of the gut microbiota at phylum, order, and genus levels. Values are presented as the means ± SD (*n* = 3). ^##^ *p* < 0.01 compared to Normal, *p < 0.05 compared to IBS.

In our study, 16S rDNA sequencing was conducted to examine whether CKF modulated gut microbiota and characterized these modifications in the composition of gut microbiota. Therefore, we sequenced the V3 + V4 region of bacterial 16S rRNA to evaluate the effects of CKF on the composition of gut microbiota. High-throughput pyrosequencing of the samples yielded 720,355 pairs of raw reads. After alignment of the pair-end reads and filtering, we obtained 718,081 clean tags for subsequent analyses. The effective reads were then clustered into OTUs based on the 97% similarity cut-off. The Shannon diversity index was higher in the IBS group related to the Normal and CKF groups (Figure 7E). Next, structural changes in the gut microbiota were evaluated using unsupervised multivariate statistical methods and principal coordinates analysis (PCoA). Results of PCoA showed that the three groups had distinct clustering for microbiota composition. Rats in the CKF group showed a structure similar to those in the Normal group (Figures 7A–D). In addition, analysis of the UPGMA clustering tree (Figure 7F) showed that the community structure of the CKF group was more similar to that of the normal group than the IBS group, indicating that CKF could significantly modulate the composition of the gut microbiota.

Further analysis of the structure of the microbial community showed a significant positive effect of CKF at both phylum, order, and genus levels. Results obtained after analysis at the phylum level showed a significant decrease in the relative abundance of *Bacteroidetes* in IBS rats and CKF treatment could restore these altered levels (Figure 7G). Moreover, the F-B ratio was higher in IBS rats as compared to normal rats, and treatment with CKF decreased the F-B ratio in IBS rats. To further examine these differences among samples, analysis was performed at the order and genus level and the results were similar to those obtained at the phylum level.

In IBS rats, analysis at the order level showed a significant increase in the relative abundances of *Clostridiales* and *Corynebacteriales* belonging to the *Firmicutes* and *Actinobacteria* phyla (Figure 7H). Notably, CKF treatment could significantly restore the relative abundances of *Clostridiales* and *Corynebacteriales*.

Analysis at the genus level showed a significant increase in the relative abundance of *Christensenellaceae_R-7_group, uncultured_bacterium_f_Ruminococcaceae, uncultured_bacterium_f_Lachnospiraceae* belonging to the *Firmicutes* phylum in IBS rats (Figure 7I). However, a decrease in the relative abundance of *Lactobacillus and Dubosiella,* belonging to the *Firmicutes* phylum, was observed in IBS rats. Notably, CKF treatment could significantly restore the relative abundance of *Christensenellaceae_R-7_group*,*uncultured_bacterium_f_Ruminococcaceae*, *uncultured_bacterium_f_Lachnospiraceae*, *Dubosiella*, and *Lactobacillus* in IBS rats.

Furthermore, the profile of gut microbiota indicated that CKF could significantly modulate the structure of the gut microbiota (Supplementary Table S2). To identify key phylotypes that were significantly altered after CKF treatment, all effective sequences were analyzed by the LEfSe method. Results of the LEfSe analysis showed the presence of high-dimensional biomarkers in the gut microbiota in each group.

**FIGURE 8:**
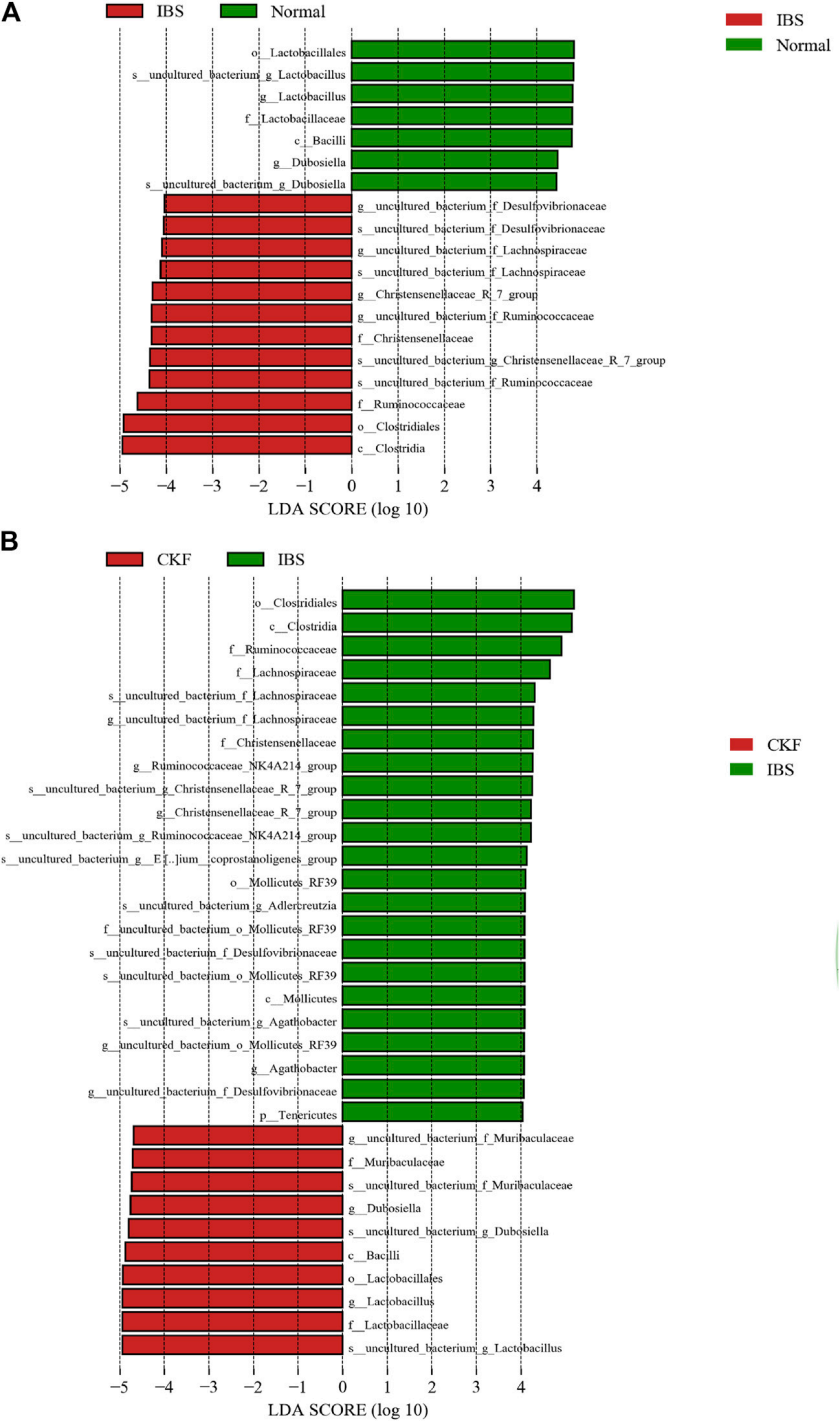
Linear discriminant analysis (LDA) plots to highlight the significantly different characteristic taxons on comparing IBS vs. Normal groups and CKF vs. IBS groups; only the taxa with a value of LDA score greater than four are shown **(A,B)**.

As shown in Figure 8A, on comparing the significant changes of gut microbiota in IBS vs. Normal groups, the dominant kinds in the IBS group were as follows: *s__uncultured_bacterium_g_Christensenellaceae*, *_R_7_group*, *g__Christensenellaceae_R_7_group*, *f__* Christensenellaceae, *s__uncultured_bacterium_f_Lachnospiraceae*, *g__uncultured_bacterium_f_Lachnospiraceae*, *s__uncultured_bacterium_f_Ruminococcaceae*, *g__uncultured_bacterium_f_Ruminococcaceae*, *f__Ruminococcaceae*, *s_uncultured_bacterium_f_Desulfovibrionaceae*, *g__uncultured_bacterium_f_Desulfovibrionaceae*, *c__ Clostridia*, and *o__Clostridiales*.

The dominant types in the Normal group were *s__uncultured_bacterium_g_Lactobacillus*, *g__Lactobacillus*, *f__Lactobacillaceae*, *o__Lactobacillales, s__uncultured_bacterium_g_Dubosiella, g__Dubosiella, s__uncultured_bacterium_g_Dubosiella, g__Dubosiella, c__Bacilli, f__Lactobacillaceae, g__Lactobacillu, s__uncultured_bacterium_g_Lactobacillus,* and *o__Lactobacillales*.

As shown in Figure 8B on comparing the significant changes in gut microbiota in CKF vs. IBS groups, the dominant types in the CKF group were identified as follows: *s__uncultured_bacterium_f_Muribaculaceae*, *g__uncultured_bacterium_f_Muribaculaceae*, *f__Muribaculaceae*, *s__uncultured_bacterium_g_Lactobacillus*, *g__Lactobacillus*, *f__Lactobacillaceae*, *o__Lactobacillales*, *s__uncultured_bacterium_g_Dubosiella*, and *g__Dubosiella*.

The dominant types in IBS group were *s__uncultured_bacterium_g_Adlercreutzia*, *s__uncultured_bacterium_g_Christensenellaceae_R_7_group*, *g__Christensenellaceae_R_7_group*, *f__*Christensenellaceae, *s__uncultured_bacterium_g_Agathobacter*, *g__Agathobacter*, *s__uncultured_bacterium_f_Lachnospiraceae*, *g__uncultured_bacterium_f_Lachnospiraceae*, *f__Lachnospiraceae*, *s__uncultured_bacterium_g_Ruminococcaceae_NK4A214_group*, *g__Ruminococcaceae_NK4A214_group*, *s__uncultured_bacterium_g__Eubacterium__coprostanoligenes*, *f__Ruminococcaceae*, *s__uncultured_bacterium_f_Desulfovibrionaceae*, *g__uncultured_bacterium_f_Desulfovibrionaceae*, *s__uncultured_bacterium_o_Mollicutes_RF39*, *g__uncultured_bacterium_o_Mollicutes_RF39*, *f__uncultured_bacterium_o_Mollicutes_RF39*, *o__Mollicutes_RF39*, *c__ clostridia*, and *o__clostridiales*.

As shown in Supplementary Table S3 on comparing the significant changes in gut microbiota between the CKF and Normal groups, the dominant types were identified, including *f__Lachnospiraceae*, *g__Lachnospiraceae_NK4A136*, *g__Eubacterium__coprostanoligenes*, *and s__uncultured_bacterium_g__Eubacterium__coprostanoligee*.

Supplementary Table S3 also suggest that organisms in the *Lactobacillus* and *Dubosiella* genus were mainly enriched in the Normal and CKF groups, whereas *Christensenellaceae_R_7_group*, *uncultured_bacterium_f_Lachnospiraceae*, *uncultured_bacterium_f_Ruminococcaceae*, *and uncultured_bacterium_f_Desulfovibrionaceae* were mainly enriched in the IBS group. Collectively, these findings suggested that CKF treatment could reverse IBS-induced dysbiosis of the gut microbiota.

### The Effects of Chang-Kang-Fang Formula on Serum Metabolites in the Treatment of Irritable Bowel Syndrome and Depression-Like Behavior

**FIGURE 9:**
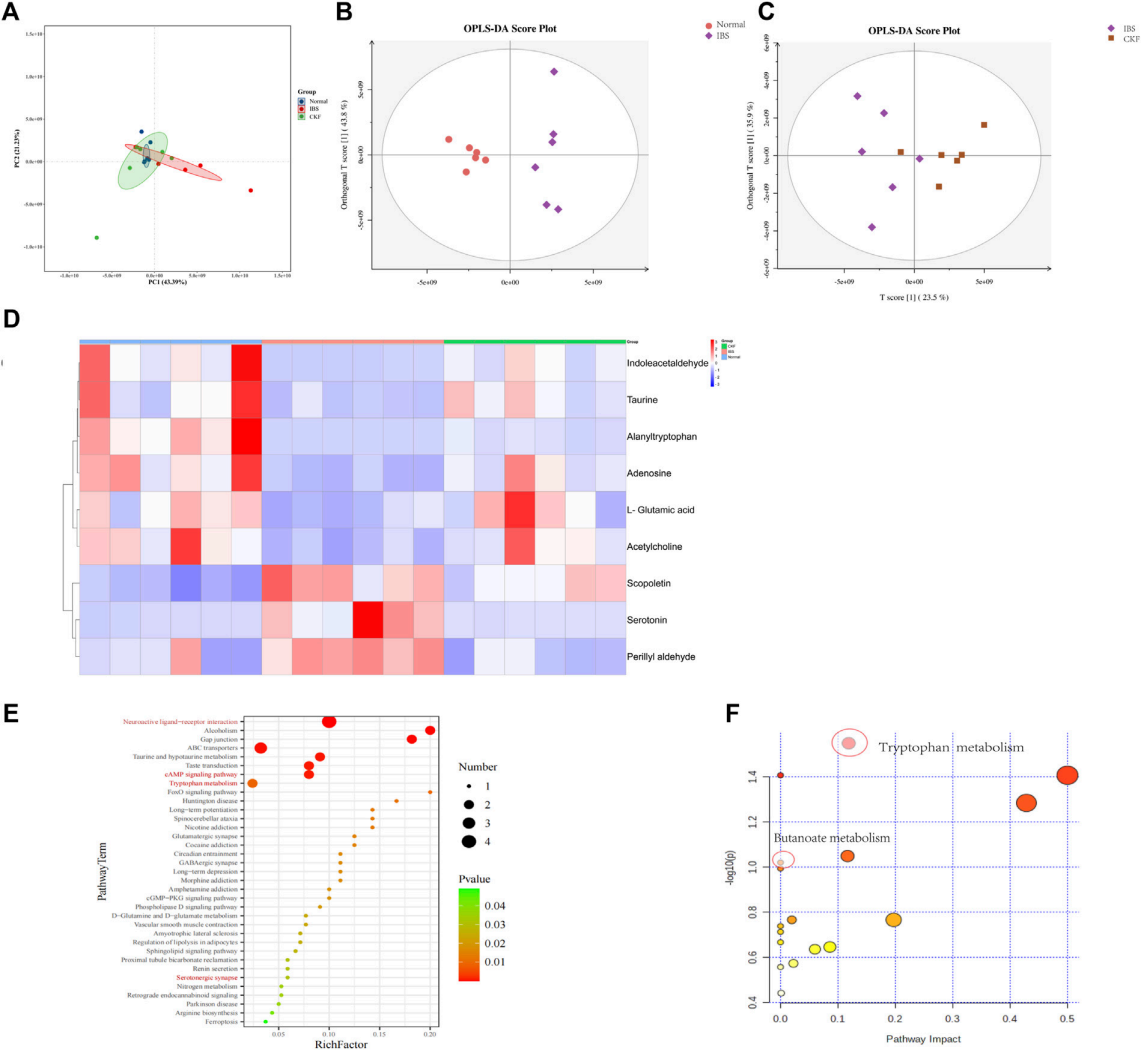
PCA & OPLS-DA score plots for Normal, IBS, and CKF groups. PCA & OPLS-DA score plots for serum comparisons **(A)** Normal, IBS, and CKF **(B)** IBS vs. Normal group **(C)** IBS vs. CKF group. **(D)** Heatmap to visualize the intensities of differential metabolites in the serum samples (*n* = 6). **(E)** KEGG pathway analysis of differentially expressed metabolites (*p* < 0.05). **(F)** Summary of pathway analyses along with metabolomics MetPA.

Metabolomics is an effective method to identify the interactions between the host and gut microbiota. Consequently, UPLC-MS/MS was used to examine serum metabolites. Serum metabolic profiling (PCA) showed that the Normal, IBS, and CKF groups could be distinguished, and the CKF group was closer to the Normal group than the IBS group (Figure 9A).

The data obtained from the serum samples were analyzed using the OPLS-DA score. The OPLS-DA score plot presented a distinct clustering of metabolites in serum samples between IBS and CKF groups as well as between the normal and IBS groups, suggesting that both IBS and CKF substantially influenced the metabolic profiles (Figures 9B,C). Following the OPLS-DA test, the prospective differential metabolites were evaluated as the integral, with a varied significance (VIP > 1.0 and *p* < 0.05) using the Student t-test. In total, 118 metabolites were significantly differentially expressed in the serum of IBS rats as compared to the normal rats (Supplementary Table S4). Treatment with CKF could reverse the levels of nine metabolites towards the normal, including L-glutamic acid, adenosine, indoleacetaldehyde, serotonin, scopoletin, acetylcholine, alanyltryptophan, perillyl aldehyde, and taurine (Figure 9D). The altered metabolites and differential metabolic pathways were identified by KEGG analysis (http://www.genome.jp/kegg/pathway.html). This analysis suggested their involvement in 50 metabolic pathways (Supplementary Table S5). Among them, metabolomics showed that amino acids (tryptophan, glutamate, taurine, hypotaurine, histidine, alanine, aspartate, glutamate, arginine, and proline), SCFAs-related (Butanoate), and 5-HT-related metabolic pathways (neuroactive ligand-receptor interaction, cAMP signaling pathway, serotonergic synapses, and inflammatory mediator regulation of TRP channels) induced by IBS were attenuated after CKF treatment. KEGG pathway enrichment analysis demonstrated that nine differentially expressed metabolites were enriched in 50 different signaling pathways; among them, 38 were significantly enriched (*p* < 0.05) (Figure 9E). To examine the possible metabolic pathways influenced by CKF, pathway analysis was performed following metabolomics using MetPA (http://www.metaboanalyst.ca/faces/home.xhtml). As shown in Figure 9F, 18 metabolic pathways were identified as important for understanding the effects of CKF on CACS-induced IBS. These results suggested that the differentially expressed metabolites in the serum of CKF and IBS rats may be associated with amino acids, SCFAs metabolism, and 5-HT related metabolic pathways. Tryptophan and butanoate metabolism were found to be the key regulatory pathways, thereby suggesting that the metabolites enriched in tryptophan and butanoate metabolism may substantially regulate the gut microbiota in CKF and IBS groups. Taken together, these findings suggested that serum metabolites effectively responded to both IBS and CKF administration and that IBS-induced serum metabolism aberrations were improved by CKF treatment.

Moreover, 36 metabolites were significantly differentially expressed in the sera of rats in the CKF group as compared to the normal group (Supplementary Table S6). The altered metabolites and differential metabolic pathways were identified by KEGG analysis. This analysis suggested their involvement in 17 metabolic pathways (Supplementary Table S7). Among them, the metabolite-related pathways were enriched in tyrosine and lactic acid metabolism pathways (Supplementary Figure S2 and Supplementary Table S7).

### Systematic Investigation of the Mechanism of Chang-Kang-Fang Formula in the Treatment of Irritable Bowel Syndrome and Depression-Like Behavior

**FIGURE 10:**
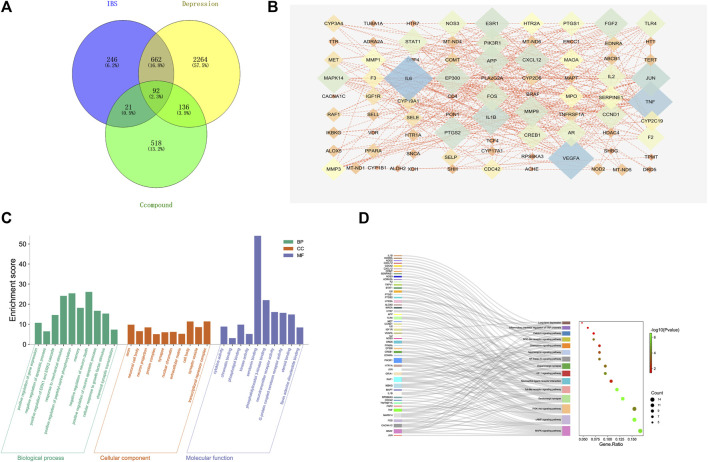
Target network analysis. **(A)** Venn diagram of intersection of the drug targets and disease proteins. **(B)** Protein-protein interaction network of target genes. **(C)** GO terms for the candidate targets of CKF in IBS; the top 10 GO functional categories are shown. **(D)** KEGG pathway enrichment analysis for the candidate targets of CKF in IBS. The sizes of the circles represent the number of genes and the color represents the significance (*p*-value).

**FIGURE 11:**
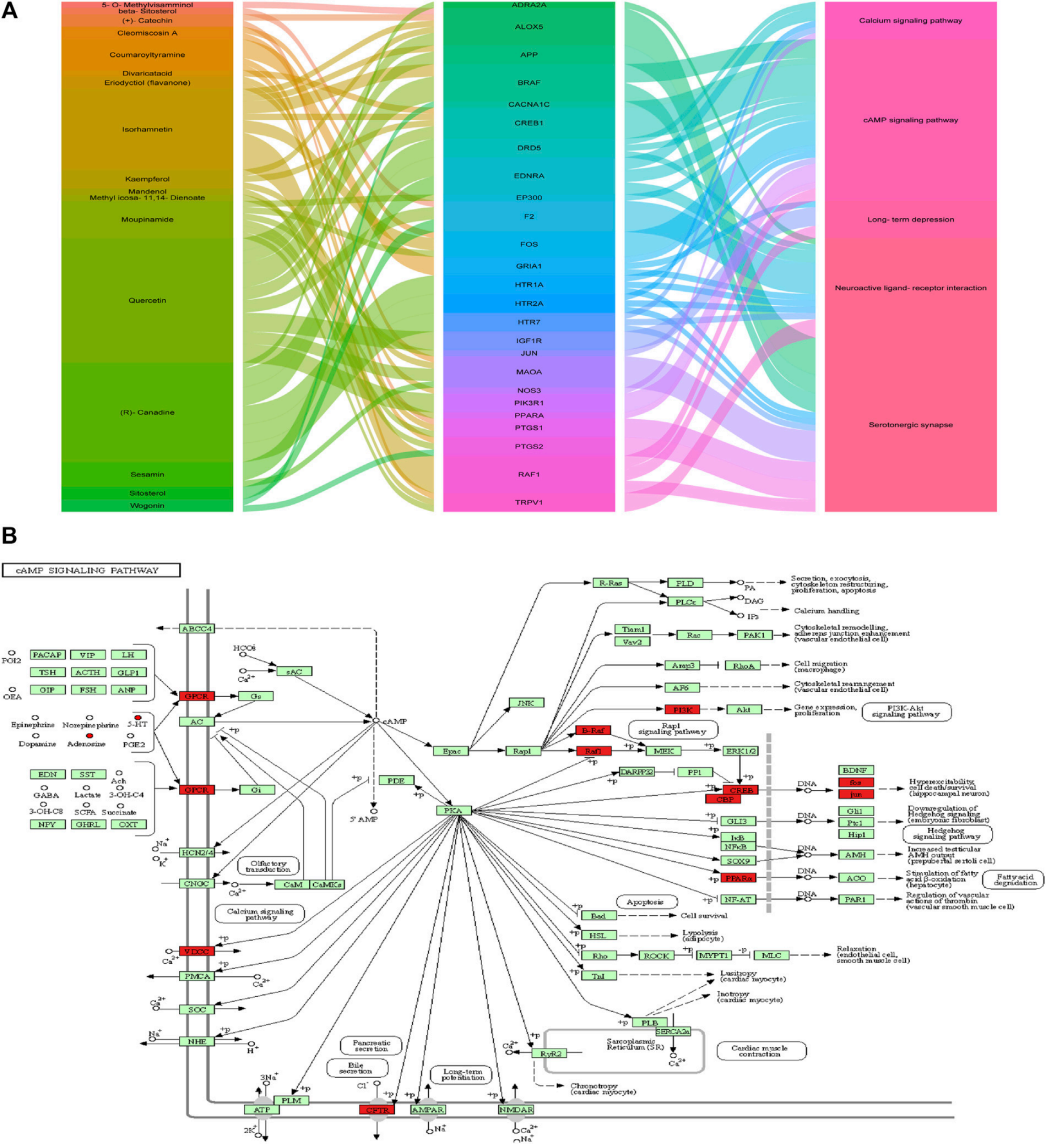
Metabolism-protein networks. **(A)** Sankey diagram of the interaction between botanical drugs of CKF, major hub genes, and main pathways. Note: the botanical drugs, major hub genes, and main pathways are shown in different colors. The height of the rectangle and the width of the connecting line are positively correlated with the number of the other rectangles that they are connected to. **(B)** Metabolism-protein networks for cAMP signaling pathway. ○ Metabolites █ Key protein targets.

A complete literature search of the CKF mix resulted in the identification of 55 chemical constituents and 767 putative protein targets, of which, 92 targets were ascertained to be associated with IBS and depression-like behaviors (Figure 10A). Next, a PPI network was constructed to examine the interaction between CKF-related hub genes in IBS and CKF-known therapeutic hub genes for IBS. The parameters for network construction were set at high confidence (interaction score ≥0.7) and the disconnected nodes were excluded from the network. The PPI network consisted of 92 nodes and 324 edges, and the two-fold median of node degree was 7.04 (Figure 10B). The major hub genes included HTR1A and CREB1. The major hub genes in the PPI network were then input into clusterProfiler for GO and KEGG analyses. A total of 436 GO terms were ascertained, including 328 biological processes (BP), 48 molecular functions (MF), and 60 cellular components (CC). The top ten terms in each category are represented in a bar chart (Figure 10C). In total, 90 KEGG pathways were identified. Among them, neuroactive ligand-receptor interaction, cAMP signaling, calcium signaling, long-term depression, and serotonergic synapses were the major pathways with the highest gene ratios and the lowest *p*-values. This suggested that they may play important roles in the CKF treatment for IBS. The top 15 KEGG pathway terms are shown in an advanced bubble chart (Figure 10D). The relationship between CKF botanical drugs, hub genes, and 5-HT related metabolic pathways (neuroactive ligand-receptor interaction, cAMP signaling pathway, calcium signaling pathway, long-term depression, and serotonergic synapses) are presented in the Sankey diagram (Figure 11A). Collectively, these results suggested that CKF may exert its therapeutic effects on IBS through multiple biological processes and pathways.

### Potential Correlations Among Phenotypes, Molecular Biology Indicators, Serum Metabolites, and Gut Microbiota

**FIGURE 12:**
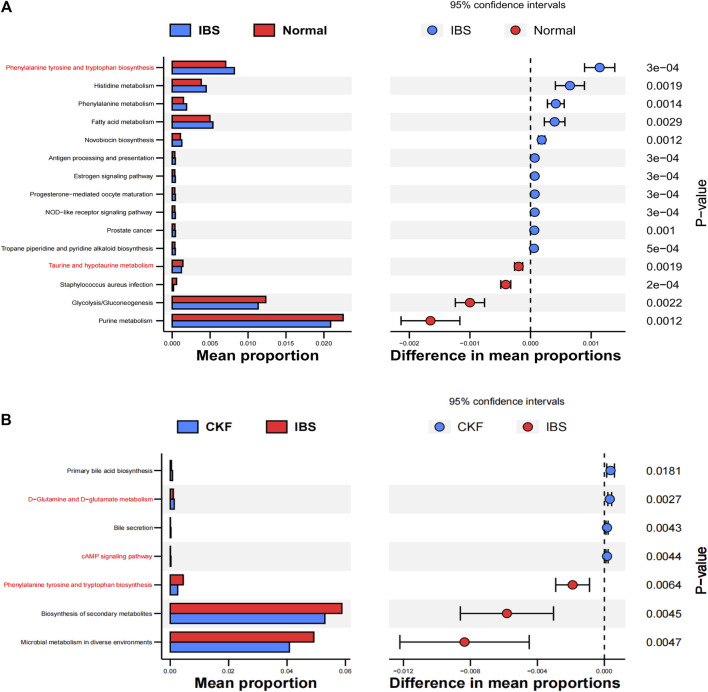
Functional predictions for the altered gut microbiota. Differential KEGG pathways on comparing IBS vs. Normal groups **(A)** and CKF vs. IBS groups **(B)**.

PICRUSt analysis based on the 16S rRNA gene sequence data was used to investigate the gut microbiome functions related to CKF treatment. The potential metabolic functions of the gut microbiota may be attributed to the alterations in bacterial taxa. The gut microbiota functions were analyzed using the KEGG database. As shown in Figure 12A, 15 different functional pathways were identified between the Normal and IBS groups. Those involved in phenylalanine, tyrosine, and tryptophan biosynthesis were upregulated, while the pathways involved in taurine and hypotaurine metabolism were downregulated in the IBS rats. CKF treatment downregulated phenylalanine, tyrosine, and tryptophan biosynthesis pathways, whereas upregulated D-glutamine and D-glutamate metabolism, along with the cAMP signaling pathway (Figure 12B). Additionally, we structured the metabolism-protein networks and identified the serum metabolites and CKF key protein targets involved in the cAMP signaling pathway (Figure 11B). The 5-HT-PKA-CREB-BDNF axis was involved in the cAMP signaling pathway. The serum metabolites and CKF key protein targets were also involved in the neuroactive ligand-receptor interaction and serotonergic synapses (Supplementary Figures S3A,B). The CKF key protein target (5-HT1A) was also involved in serotonergic synapses. The key serum metabolites (l-glutamate, taurine) were also found to participate in neuroactive ligand-receptor interactions.

The relationships among host phenotypes, serum metabolites, and the gut microbiota were comprehensively analyzed. The correlation matrix was generated by calculating Spearman’s correlation coefficient (Supplementary Figure S4 and Supplementary Table S8). In the correlation between serum metabolites and gut microbiota, *uncultured_bacterium_f_Lachnospiraceae* were found to be negatively correlated with tryptophan-related metabolites (indoleacetaldehyde), neuroactive ligand-receptor interaction-related metabolite (taurine), and butanoate-related metabolite (L-glutamic acid), whereas two bacterial genera, including *Dubosiella* and *Lactobacillus*, showed a positive correlation with the corresponding aforementioned serum metabolites. *Clostridiales* was found to be negatively correlated with indoleacetaldehyde and taurine and *Corynebacteriales* was found to be positively correlated with serotonin.

For gut microbiota and phenotypes, 20 mmg AWR Score and GI transit were found to be negatively correlated with *Dubosiella* and *Lactobacillus*, whereas positively correlated with *Clostridiales* and *uncultured_bacterium_f_Lachnospiraceae*. Sucrose Preference were found to be positively correlated with *Dubosiella* and *Lactobacillus*, whereas negatively correlated with *Clostridiales, uncultured_bacterium_f_Ruminococcaceae* and *uncultured_bacterium_f_Lachnospiraceae*. *Clostridiales and Corynebacteriales* were found to be positively correlated with FST immobility time.

For gut microbiota and indicators of molecular biology, including the concentrations of 5-HT and BDNF in the colon were found to be negatively correlated with *Lactobacillus*, whereas positively correlated with 5-HT and BDNF concentrations, the mRNA expressions of 5-HT1A, BDNF, PKA, and CREB in the hippocampus. 5-HT and BDNF concentrations in the colon were positively correlated with *Clostridiales* and *uncultured_bacterium_f_Lachnospiraceae*, whereas negatively correlated with 5-HT and BDNF concentrations, the mRNA expressions of 5-HT1A, BDNF, PKA, and CREB in the hippocampus. The gut microbiota could thus significantly affect the host phenotypes, molecular biology indicators, and metabolite concentrations.

## Discussion

IBS is a disease characterized by abdominal pain, which is usually accompanied by bloating and changes in bowel habits (Liu et al., 2021). Similar to other dysfunctions, IBS is associated with a high incidence of mental illness, with some studies reporting that approximately 90% of IBS patients suffer from anxiety and major depression (MDD) (Lydiard, 2001; Friedrich et al., 2010). Generally, it is known that the gut function is regulated by the central nervous system. In reflex regulation and emotional control, signals from sensory sources influence the brain. Memory and cognition due to stress alter the inputs, which are then integrated into neural circuits of the CNS, autonomic nervous system (ANS), and enteric nervous system (ENS) (Kirkup et al., 2001; Drossman et al., 2002). Animal models of chronic and acute stress have been extensively utilized to unravel the biological mechanisms underlying affective and somatic disorders. Animals with CACS have emotional disorders, abnormal intestinal motility, and visceral hypersensitivity (Mineur et al., 2007; Ping et al., 2014; Yu et al., 2015; Moloney et al., 2016; Xu et al., 2018). The findings of the present study suggested that CACS induction in rats for 22 days resulted in a series of abnormalities both affective and somatic dysfunctions, i.e., the occurrence of diarrhea, depression- and anxiety-like behaviors, gastrointestinal hypomotility, and hypersensitivity, thereby mimicking the symptoms of patients with IBS.

In addition, IBS models show various phenotypic changes, indicating the presence of a combination of depression and functional bowel disease. Several studies show that IBS can trigger depression-like behaviors. Results showed that the sucrose preference ratio reduced and immobility time in the FST increased in IBS rats (Ness and Gebhart, 1988; Yu et al., 2015; Xu et al., 2018; Yu et al., 2019). AWR is a semi-quantitative method for measuring the involuntary movement reflex in response to visceral pain. The CACS model showed increased visceral toxicity to CRD at different intensities, consistent with previous reports on visceral hyperalgesia in an animal model of IBS (Al-Chaer et al., 2000; Yu et al., 2012). It reported that processes underlying chronic and acute stress can mimic the pathophysiological changes in IBS (Zou et al., 2008). The tests conducted in this study demonstrated the impact of CKF on IBS. The CACS animal model showed multiple phenotypic changes, indicating the coexistence of depression and functional bowel disease. Given the results, to the best of our knowledge, we are the first to determine whether CKF can improve IBS-like behavior to an extent comparable to TM. Indeed, TM, an antispasmodic drug, could improve the peripheral dysfunctional symptoms of IBS more effectively and the weights of the rats were closer to the normal group, visceral sensitivity, total fecal output, fecal water content, and intestinal operation were comparable to the CKF group. In terms of antidepressant-like behaviors (SPT and FST), 5.0 g/kg CKF was closer to the normal group than the TM group. CKF not only reduced CACS-induced intestinal sensitivity, like in bowel movement and CRD behavioral tests but also triggered antidepressant effects, as shown by the results of the SPT and FST. These findings suggested that CKF treatment could reverse IBS-induced intestinal sensitivity and depression-like behaviors. Additionally, we conducted preliminary experiments (*n* = 6), and the antidepressant-like behaviors at a medium dose (2.5 g/kg) of CKF were not statistically significant as compared to those in the IBS group (Supplementary Table S9).

As shown in Figure 3, 5-HT is mainly found in the enterochromaffin cells (EC) in the intestinal endothelium; to a lesser extent, it is present in the intermuscular and submucosal plexus of the ENS. Mutual brain-gut interactions, including the 5-HT pathway, significantly affect the effector system. 5-HT plays a critical role in the intestines, where it regulates movement and sensory events in the GI tract by activating different 5-HT receptors, which are also emotional conductors in the hippocampus. Our findings suggested that 5-HT concentration markedly reduced in the hippocampus of the IBS rats in comparison to the normal group, however, that in the colon increased. Visceral hyperalgesia in rats with early stress is associated with the increase in 5-HT expression in colonic stress responses. Serotonergic neurons are located in the median raphe nucleus, which affects the spinal cord and brainstem and participates in the central pain regulation (Gershon and Tack, 2007). 5-HT1A receptors have received great attention among the serotonin receptor subtypes for the treatment of IBS as they modulate the pathogenesis of depression and the anti-depression responses (Bambico et al., 2009). A previous group also hypothesized that they mediate visceral sensitivity in the gut (Bueno and Fioramonti, 2002). Results obtained in this study suggested that CKF can alleviate hyperalgesia and antidepressant-like behaviors by modulating the 5-HT system in a rat model of CACS. Given that altered cAMP signaling is associated with major depression and GI dysfunction (Cowburn et al., 1994), it is a potential target for the treatment of IBS symptoms (Xu et al., 2018; Yu et al., 2019). Notably, cAMP results in an increased sensitivity of the cAMP-dependent protein kinase (PKA), thereby triggering downstream cAMP signaling proteins such as CREB. Phosphorylation of CREB modulates the transcriptional activity and enhances the expression of BDNF, resulting in antidepressant and neuroprotective effects (Reichardt, 2006; Yossifoff et al., 2008). The BDNF gene contains the cAMP response element (CRE), which binds to phosphorylated CREB, thereby increasing its transcription. Moreover, elevated BDNF in the colon, caused due to increased colorectal dilatation, has also been observed in IBS patients (Yu et al., 2012). Interestingly, the levels of 5-HT1A, PKA, CREB, and BDNF decreased significantly in the hippocampus of IBS rats, while elevated expressions were observed in their colons. This finding suggested that psychological stress could indirectly induce excessive arousal of the colon by inactivating the CNS. After CKF treatment, the levels of 5-HT, 5-HT1A, PKA, CREB, and BDNF increased in the hippocampus and decreased in the colon, thereby indicating that CKF can alleviate the symptoms of IBS rats by modulating the brain-gut axis through the 5-HT1A-PKA-CREB-BDNF pathway.

To study the change in microbiota composition, a 16S rRNA sequencing was also performed. The results suggested an increase in the alpha diversity of gut microbiota in the IBS group, having a higher Shannon index as compared to the normal group. Nevertheless, CKF treatment decreased the diversity. In a previous study on the gut-microbiota-brain axis involving IBS patients, higher alpha diversity was found in IBS patients, and for the first time, through this study, the brain structural alterations associated with IBS subgroups and the correlations of the abundance of certain microbial taxa with early psychosocial stress were identified (Labus et al., 2017). A study shows that fecal microbiota diversity is rich in IBS-D patients relative to the healthy group and there are alterations in *Bacteroidales* and *Clostridiales* at the order levels. As *Bacteroidales* and *Clostridiales* may be involved in the pathophysiology of IBS-D, in our study, IBS was found to be enriched in *Clostridiales* as compared to both Normal and CKF groups S (Zhuang et al., 2018). Some studies indicate that beneficial bacterial species (*Lactobacillus*) are associated with a reduction in harmful bacteria (*Clostridium*) (Distrutti et al., 2016). Among the differential markers in our study, IBS was enriched in *Clostridiales* (order) as compared to both Normal and CKF groups, while Normal and CKF were enriched in *Lactobacilli* relative to IBS (genes).

Moreover, PCoA and system clustering trees showed substantial distances between each group, indicating that the beta diversity of gut microbiota in the CKF group differed significantly from that in the IBS group. Based on recent studies, a high ratio of *Firmicutes* to *Bacteroidetes* (F-B ratio) is closely associated with the IBS group. Herein, the findings suggested that the F-B ratio was higher in the IBS group relative to the normal rats, and CKF treatment decreased the F-B ratio in the IBS rats. However, the results also showed that the relative abundance of *Lactobacillus* was lower in the IBS group relative to the normal rats. Moreover, CKF treatment increased the relative abundance of *Lactobacillus*, which has been previously demonstrated to exert beneficial clinical effects against the IBS (Fan et al., 2006; Sinn et al., 2008).

SCFAs, in particular, butanoate, can increase the production of *Lactobacillus* and are the main metabolites of the gut microbiota (Pouteau et al., 2003; Dalile et al., 2019). Abnormal changes in SCFAs are associated with IBS as they are the key signaling molecules that regulate intestinal functions (Zhu et al., 2019). Furthermore, the imbalance in the gut microbiota of IBS patients directly affects the normal signaling interactions among the gut microbiota, SCFAs, and intestinal epithelial cells (Zhu et al., 2019). *Lactobacillus* may also modulate cognitive impairment, anxiety, and depression because it has positive impacts on the CNS through the modulation of neuroinflammation (Paiva et al., 2020). *Lactobacillus* strains influence CNS-related functions and behaviors mediated by the MGBA *via* immune, humoral, neural, and metabolic pathways to improve intestinal functions and antidepressant effects (Cheng et al., 2019).

We found that 5-HT pathways are also involved in the bidirectional MGBA interactions with profound effects on the effector systems (Figure 3), and a change in the microbiota could improve 5-HT-related disease symptoms (Yano et al., 2015). Interestingly, the indigenous microbiota also modulates hippocampal 5-HT levels by influencing the availability of tryptophan, the 5-HT precursor, suggesting a role of the microbiota in regulating the serotonergic system of the brain (Clarke et al., 2013; Yano et al., 2015). Several previous studies report that ingestion of *Bifidobacteria* or *Lactobacilli* beneficially disrupts either anxiety or depression-like behaviors, both under pathological conditions and in healthy animals (Desbonnet et al., 2010; Bravo et al., 2011; Messaoudi et al., 2011). A previous study shows that *Lactobacillus* both decreased the abundance of *Firmicutes*, *Clostridia*, and *Clostridiales*, along with a marked decrease in the level of colonic 5‐HT as compared to the rat model with chronic unpredictable mild stress (Li et al., 2019). At the order level, IBS was enriched in *Clostridiales* (order) as compared to Normal and CKF groups, and in *Corynebacteriales*. In the CKF group, the abundance of *Clostridiales* (order) and *Corynebacteriales* (order) decreased significantly. *Corynebacterium* spp. directly produces serotonin in the gut (Xiao et al., 2021). *Clostridium*, belonging to the genus of *Firmicutes* and *Clostridia*, including 221 types, produces tryptamine prompting colonic 5-HT biosynthesis (Parte, 2014; Xiao et al., 2021).

Further, we analyzed the serum metabolites, since these were the products of metabolic activity of the host and microbiome, and the results showed that the CKF significantly affected the serum metabolite levels, including amino acids (tryptophan, glutamate, taurine, hypotaurine, histidine, alanine, aspartate, glutamate, arginine, and proline), SCFAs-related metabolism (butanoate), and 5-HT related metabolic pathways (neuroactive ligand-receptor interaction, cAMP signaling pathway, and serotonergic synapses). In particular, CKF treatment altered the concentrations of serotonin, indoleacetaldehyde, taurine, and L-glutamic acid. Further, the results showed that the IBS model was associated with a reduction in serum indoleacetaldehyde, taurine, and L-glutamic acid levels, while CKF treatment could significantly elevate these levels. We also examined the correlation of CKF with serum metabolites and gut microbiota. Indoleacetaldehyde, taurine, and L-glutamic acid were negatively correlated with the *Dubosiella and Lactobacillus* genera. *Clostridiales* were negatively correlated with Indoleacetaldehyde and taurine, whereas *Corynebacteriales* positively correlated with serotonin. These findings suggested that the gut microbiota influenced serum metabolism. Furthermore, the possible biomarkers related to gut microbiota were examined to better understand the therapeutic value of CKF which could modulate the microbiota-gut-brain axis *via* 5-HT-BDNF related pathways, and for IBS and depression-like behaviors. Recently, several studies have shown that the gut microbial species produce multiple tryptophan catabolites through different metabolic pathways (Figure 3). Indoles and indole derivatives, such as indole-3-aldehyde, indole-3-acetaldehyde, and indoleacrylic acid, are bioactive substances produced directly by the activity of tryptophanase in *Escherichia coli* and *Lactobacillus* (Hubbard et al., 2015). Disorders due to tryptophan metabolism are caused by the gut microbiota, and thus, the intermediate tryptophan (5-HT) can activate the brain-gut nerve responses, leading to diarrhea (Spencer and Hu, 2020; Ming et al., 2021). *Clostridium* converts tryptophan into tryptamine, indolelactic acid (ILA), and indolepropionic acid (IPA) (Roager and Licht, 2018). In enterochromaffin cells, tryptamine induces the release of 5-HT. Moreover, natural taurine reduces the abundance of *Clostridia* (Yu et al., 2016). The functional capabilities of the microbial communities were analyzed by PICRUSt. The pathways involved in phenylalanine, tyrosine, and tryptophan biosynthesis were upregulated, whereas taurine and hypotaurine metabolism were downregulated in the IBS rats. Thus, the increase in *Clostridium* abundance may be associated with tryptophan biosynthesis and taurine metabolism in IBS rats. Additionally, pathways involved in the cAMP signaling pathway and D-glutamate metabolism were also upregulated, whereas phenylalanine, tyrosine, and tryptophan biosynthesis were downregulated in the CKF group. The reduction in the abundance of *Clostridium* may downregulate tryptophan biosynthesis and taurine metabolism, while the increase in *Lactobacillus* may upregulate the cAMP signaling pathway and D-glutamate metabolism in the CKF group. We plan to examine the correlation between the metabolites and gut microbiota by fecal transplantation assays in the future.

Additionally, the differential gut microbes and metabolites of Normal vs. CKF groups were analyzed. Our results suggested that *Lachnospiraceae_NK4A136*,*_Eubacterium__coprostanoligenes* was enriched in the Normal group, whereas the proportion of *Lactobacillus* in the CKF group was greater relative to the Normal group. Moreover, different metabolites were enriched in tyrosine and lactic acid metabolism pathways. *Lactobacillus* can influence tyrosine and lactic acid metabolism (Pessione, 2012). Thus, CKF relieving IBS may not simply restore the gut microbiota to normal levels. Indeed, CKF may markedly increase the proportion of *uncultured_bacterium_g_Lactobacillus*.

System pharmacology provides easy paradigms in traditional Chinese medicine research to assess the multi-target influences of drug action and theoretical perception underlying biological network functioning (Ren et al., 2014; Yu et al., 2015). To better understand the molecular modes of the therapeutic effects of CKF on IBS and depression-like behaviors, six pathways closely associated with 5-HT-BDNF were used to evaluate the relationship among the components, targets, and pathways. We identified 17 chemical ingredients and 25 molecular targets. This suggested that multiple targets of the same chemical or different chemicals having the same target can more critically influence the entire network balance, thereby improving the efficacy of CKF treatment. In addition, the 25 molecular targets included CREB1 and 5-HT1A, which are known to be closely associated with the 5-HT-PKA-CREB-BDNF-related pathways. These findings were consistent with those of the serum metabolome-associated pathways which demonstrated the associated pathways enriched with 5-HT-related metabolic pathways (neuroactive ligand-receptor interaction, cAMP signaling pathway, and serotonergic synapses). L-glutamate and taurine were also involved in neuroactive ligand-receptor interaction.

The results obtained in this study show the therapeutic potential of the CKF mixture in improving IBS and depression-like behaviors. Treatment with CKF significantly ameliorated the above-mentioned central nervous and peripheral symptoms in our model rats, and these effects could be attributed to the differential regulation of 5-HT in the brain vs. intestine. Thus, the comparison between IBS and CKF groups showed the “preventative” amelioration effects of CKF for IBS in the CACS animal model. Our results demonstrated that CKF significantly improved CACS-induced psychiatric and gastrointestinal dysfunctions, which further showed the “preventative” therapeutic value of CKF for CNS disorders related to the IBS symptoms. The mechanism underlying CKF effects was examined using the LC-MS-based metabolomic approach in combination with multivariate data analysis. CKF was found to play a regulatory role in mediating the concentrations of different metabolites, including amino acids, SCFAs, and the metabolites of the 5-HT-related metabolic pathways. The metabolite biomarkers were used to identify CKF mixture-related proteins and the 5-HT-PKA-CREB-BDNF associated pathways. The retrieved pathways showed a close association of the CKF mixture with IBS and depression-like behaviors. Collectively, the results obtained in this study suggested that CKF treatment induced structural changes in the gut microbiota, thereby regulating it by decreasing the F-B ratio, and the abundances of *Corynebacteriales* and *Clostridiales*, whilst increasing the levels of *Lactobacillus*. *Lactobacillus* may regulate the 5-HT pathway in the hippocampus through tryptophan metabolism by humoral circulation, thereby providing beneficial effects against intestinal tract motility, high visceral sensitivity, and behavioral abnormalities through the microbiota-gut-brain axis. Notably, phytotreatment resulted in the enrichment of useful bacteria by decreasing *Clostridiales* abundances in the gut. Moreover, CKF regulated the 5-HT1A-PKA-CREB-BDNF axis in the colon and hippocampal tissues along with the expression of the relevant pathway-related genes.

## Data Availability

The original contributions presented in the study are publicly available. This data can be found here: PRJNA765128.

## Supplementary Material

Supplementary Figure S1 Total ion currents (TIC) chromatograms of the major constituents in CKF. The compounds are detected under positive **(A)** and negative **(B)** mode, respectively.

Supplementary Figure S2 Schematic diagram of the relationship between metabolic pathways and metabolite identified by the analysis of serum samples in Normal and CKF group.

Supplementary Figure S3 Metabolism-protein networks for neuroactive ligand-receptor interactions **(A)** and serotonergic synapses **(B)**. ○ Metabolites █ Key protein targets.

Supplementary Figure S4 Association map for the four-tiered analyses integrating the gut microbiome, phenotypes, molecular biology indicators, and serum metabolites. The intensities of the colors represent the degree of association, (red) positive correlation or (blue) negative correlation. |r| ≥ 0.5, **p* < 0.05.
