# Supplementary figures and images for: Effects of Chang-Kang-Fang Formula on the Microbiota-Gut-Brain Axis in Rats With Irritable Bowel Syndrome
Source: Front Pharmacol. 2022 May 9;13:778032. doi: 10.3389/fphar.2022.778032 (PMC9125359; doi:10.3389/fphar.2022.778032)

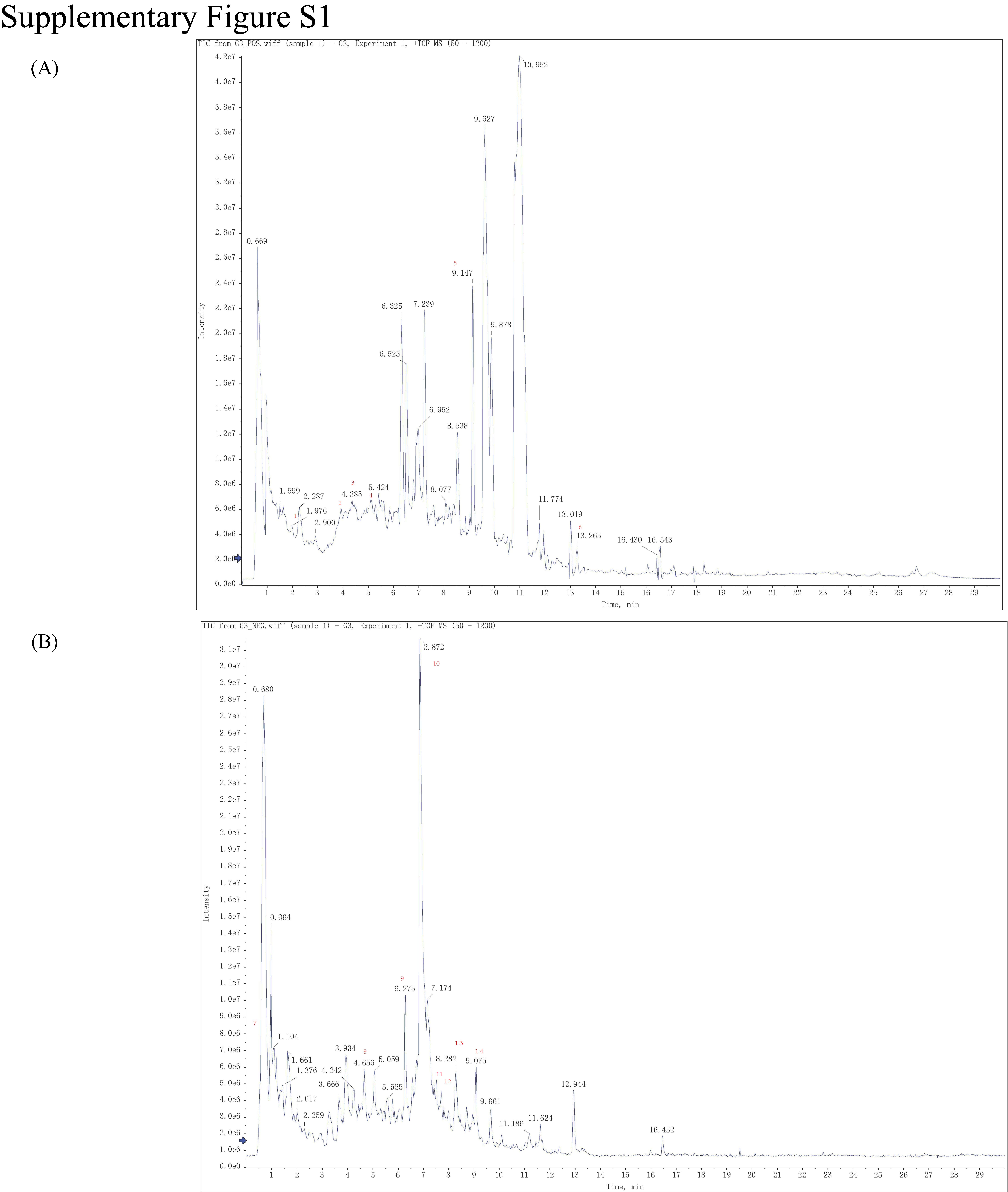

Supplement: Supplementary file 1 [file Presentation1.zip › Supplementary Figure S1.tif]

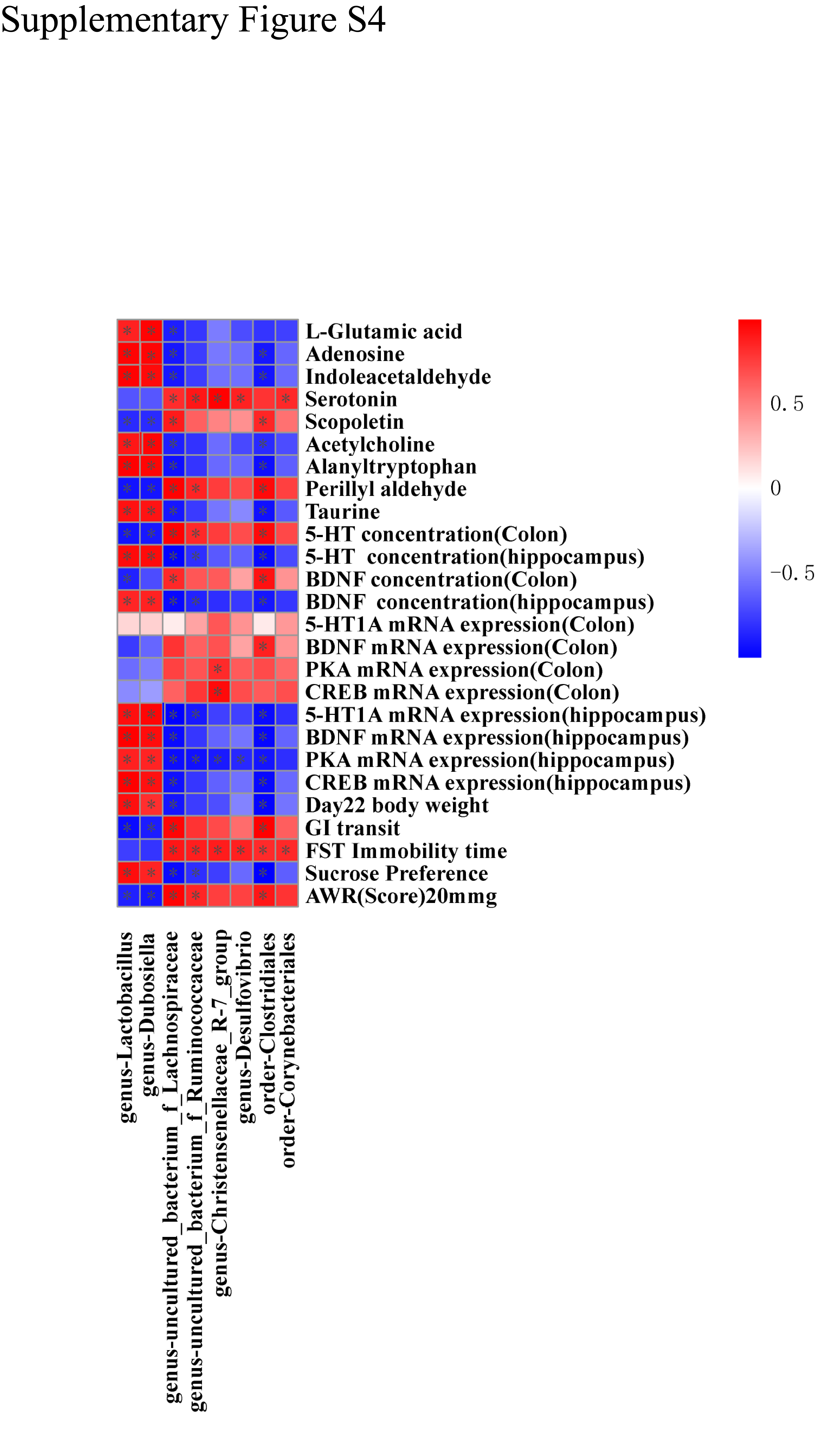

Supplement: Supplementary file 1 [file Presentation1.zip › Supplementary Figure S4.tif]
