# Supplementary material for: Effects of Chang-Kang-Fang Formula on the Microbiota-Gut-Brain Axis in Rats With Irritable Bowel Syndrome
Source: Front Pharmacol. 2022 May 9;13:778032. doi: 10.3389/fphar.2022.778032 (PMC9125359; doi:10.3389/fphar.2022.778032)
Supplement: Supplementary file 1 [file Presentation1.zip › Supplementary Table 9.doc]

**Supplementary Table S9 Middle, high doses of CKF alleviate the symptoms of IBS rats in preliminary experiment**. Values are presented as the means ± SD (n=6). ^###^*p* < 0.001 compared to Normal, **p* < 0.05, ***p* < 0.01 and ****p* < 0.001 compared to IBS.

| Group (n=6) | | Normal | IBS | TM | CKF-L  1.3g/kg | CKF-M  2.5 g/kg | CKF-H  5.0 g/kg |
| --- | --- | --- | --- | --- | --- | --- | --- |
| Body weight  (g) | Day 0 | 208.27±16.95 | 209.87±18.98 | 182.42±1.42 | 196.48±4.81 | 200.23±14.78 | 202.13±15.79 |
|  | Day 22 | 375.22±22.30 | 293.53±19.39### | 351.95±24.54*** | 310.67±11.84 | 335.77±12.23** | 336.70±12.12** |
| Food intake  (g) | Day 0 | 21.2±0.92 | 22.27±1.12 | 22.43±1.19 | 21.42±2.11 | 21.65±0.95 | 22.33±1.05 |
|  | Day 22 | 24.43±1.21 | 19.82±1.03### | 23.00±0.90* | 20.37±0.77 | 23.33±2.41** | 28.73±2.16*** |
| Stool water content (×100%) | | 0.33±0.05 | 0.78±0.08### | 0.32±0.06*** | 0.61±0.11** | 0.43±0.04*** | 0.43±0.06*** |
| GI transit  (×100%) | | 0.58±0.02 | 0.69±0.03### | 0.51±0.02*** | 0.67±0.02 | 0.64±0.02** | 0.53±0.02*** |
| AWR(Score)20mmg | | 1.33±0.52 | 3.67±0.52### | 1.17±0.41*** | 3.33±0.52 | 2.50±0.55** | 2.33±0.52** |
| FST Immobility time  (s) | | 56.50±20.46 | 143.66±30.96^###^ | 99.00±8.60^*^ | 132.00±20.98 | 116.00±9.40 | 62.50±26.04^**^ |
| Sucrose Preference  (×100%) | | 0.94±0.04 | 0.77±0.07^###^ | 0.91±0.05*** | 0.73±0.02 | 0.77±0.08 | 0.93±0.02*** |
